# Magnetically Reconfigurable Mode Strong Coupling Toward Tunable Nanophotonic Sensing Devices

**DOI:** 10.1002/nap2.70174

**Published:** 2026-06-17

**Authors:** Runmin Liu, Shengyi Wang, Liangui Deng, Hua Ge, Qiu Wang, Bowen Jia

**Affiliations:** ^1^ School of Information Engineering Wuhan University of Technology Wuhan China; ^2^ School of Physics and Technology Wuhan University Wuhan China

**Keywords:** magneto‐optical metasurface, quasi‐bound states in the continuum, refractive‐index sensing, strong coupling

## Abstract

Dynamic sensing is an important goal in nanophotonics, yet many tunable sensing platforms still rely on structural reconfiguration or material‐property switching, which can limit operational stability, integration simplicity, and real‐time controllability. Here, we present a magneto‐optical metasurface that enables tunable strong coupling for reconfigurable refractive‐index sensing through noncontact magnetic‐field control without altering the device geometry. The system supports strong coupling between a structural quasi‐bound state in the continuum (q‐BIC) induced by symmetry breaking and a magneto‐structurally induced q‐BIC, termed the MS‐BIC, giving rise to upper and lower hybrid branches whose spectral positions and Rabi splitting can be actively controlled by an external static magnetic field. For effective refractive indices of the analyte‐loaded porous environment ranging from n=1.01 to 1.15, the upper and lower branches exhibit sensitivities of about 479 and 461nm/RIU, respectively, whereas the lower branch reaches a figure of merit as high as $915\;{RIU}^{-1}$. Moreover, magnetic‐field tuning introduces multiple addressable resonance features under a fixed analyte condition, providing redundant observables for cross‐validated and noise‐robust sensing. These results establish magnetically reconfigurable strong‐coupling metasurfaces as a promising platform for high‐performance refractive‐index sensing in nanophotonic systems.

## Graphical abstract

A magnetically reconfigurable metasurface realizes tunable strong coupling between structural and magneto‐structurally induced q‐BIC resonances without changing device geometry. The resulting hybrid branches enable high‐sensitivity refractive‐index sensing with noncontact active control, large figure of merit, and multiple resonance observables, offering a promising route toward dynamic nanophotonic sensing.

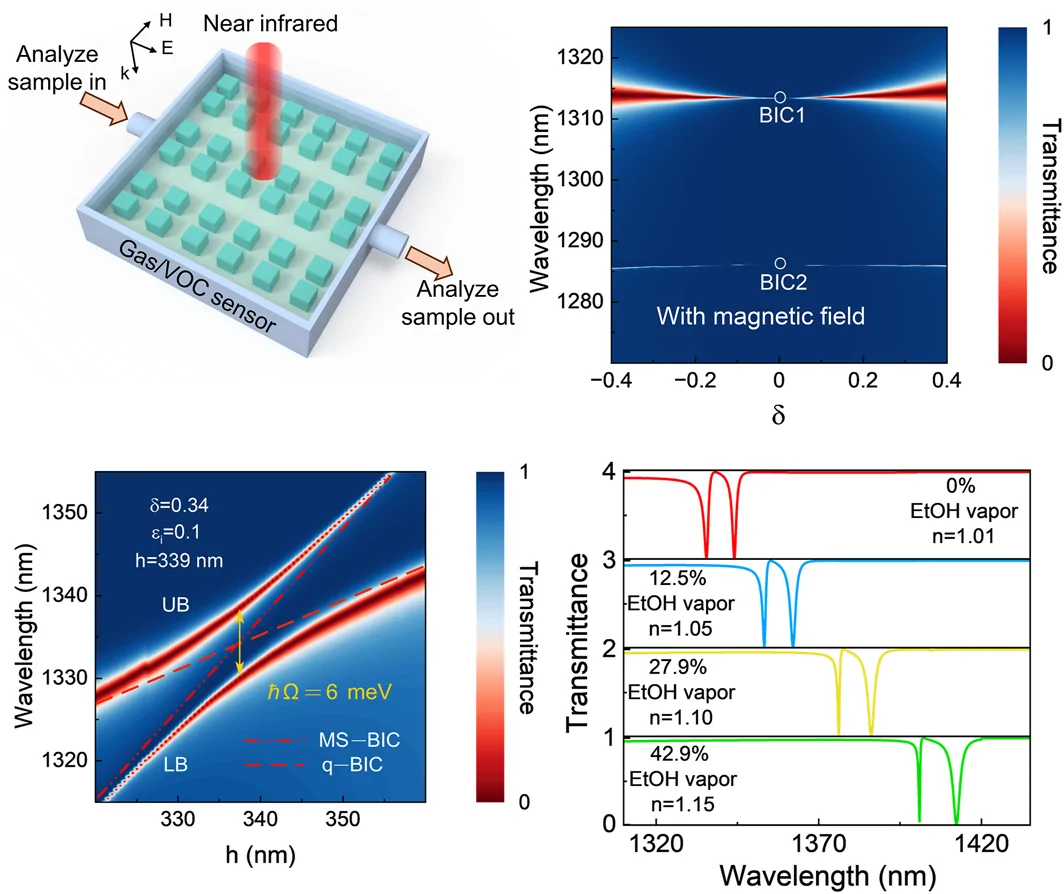

## 1 Introduction

Dynamic sensing has emerged as an important direction in nanophotonics because it enables real‐time optical responses to environmental changes and opens new possibilities for reconfigurable detection, adaptive identification, and multiparameter sensing. Compared with conventional optical sensors, dynamically tunable platforms can provide adaptive operating windows and repeated interrogation under different spectral states. To achieve dynamically tunable optical functionalities, most existing approaches rely on either material‐state variation or structural reconfiguration. Phase‐change materials such as GST, GSST, and ${\text{VO}}_{2}$ provide pronounced changes in optical constants through phase transitions, whereas active media such as liquid crystals, graphene, and ITO typically modulate resonant behavior through molecular reorientation or carrier‐density tuning [1, 2, 3, 4]. In addition to tuning based on changes in material properties, mechanical reconfiguration represents another important strategy for dynamic metasurfaces, enabling tunability by changing the geometry or relative positions of resonant elements through stretching, electrostatic actuation, thermally induced deformation, or MEMS/NEMS‐based schemes [5]. Recent representative studies have demonstrated both the richness and the limitations of these dynamic platforms. For example, Cotrufo et al. realized reconfigurable image processing in a nonlocal phase‐change metasurface by exploiting the temperature‐driven transition of ${\text{VO}}_{2}$ [6]. Sisler et al. demonstrated electrically tunable space–time metasurfaces at optical frequencies [7]. Ma et al. further achieved electrically switchable multichannel wavefront control by combining cascaded metasurfaces with liquid‐crystal phase retarders [8], whereas Siegel et al. reported electrostatic steering of thermal emission by integrating graphene with a metasurface supporting delocalized modes [9]. Together with related developments, these studies establish dynamic metasurfaces as versatile platforms for reconfigurable nanophotonic devices, including tunable q‐BIC and refractive‐index sensing systems [10, 11]. However, they also highlight a central challenge: many existing tuning strategies still rely on structural variation, lossy active media, or relatively complex driving schemes, whereas sensing platforms that preserve a fixed device geometry while enabling continuous and reversible control remain less explored. This issue is particularly important for nanophotonic sensing because repeated geometric modification or strongly lossy tuning media may deteriorate spectral sharpness, reduce device stability, and complicate integration. Therefore, developing a dynamically tunable sensing platform that maintains a fixed geometry while allowing continuous and reversible control remains an important challenge in nanophotonics.

Among various nanophotonic sensing mechanisms, platforms based on bound states in the continuum (BICs) and their radiative counterparts, quasi‐BICs (q‐BICs), have attracted particular attention because of their high‐Q resonances, strong near‐field confinement, and sharp spectral features, all of which are advantageous for high‐performance refractive‐index sensing [12, 13, 14, 15, 16]. In dielectric metasurfaces, structurally induced q‐BIC resonances have been widely exploited to achieve high sensitivity and spectral resolution [17, 18, 19]. The appeal of q‐BIC platforms lies not only in their narrow linewidths, but also in their ability to provide strong field enhancement and modal selectivity within compact subwavelength architectures, enabling applications such as narrowband spectral shaping, enhanced nonlinear generation, low‐threshold lasing, and tailored emission control [20, 21, 22, 23, 24, 25, 26]. These properties are particularly attractive for sensing, where small environmental perturbations must be converted into measurable spectral shifts while preserving a sufficiently narrow resonance linewidth for accurate readout [27, 28, 29]. Recent studies have further confirmed the sensing potential of q‐BIC metasurfaces. For instance, Watanabe et al. reported nanogap‐enhanced refractometric sensitivity in all‐dielectric q‐BIC metasurfaces [30], Romano et al. demonstrated label‐free sensing of ultralow‐weight molecules using all‐dielectric metasurfaces [31], and van Loon et al. reported refractive‐index sensing in silicon metasurfaces supporting multiple q‐BIC resonances induced by broken symmetries [32]. Li et al. subsequently demonstrated an ultrasensitive all‐dielectric q‐BIC metasurface sensor based on symmetry‐broken amorphous‐silicon pillars [33]. Despite these advances, most existing q‐BIC sensing schemes remain essentially nontunable: once the device geometry is fixed, the resonance wavelengths, linewidths, and modal properties are largely predetermined so that a given analyte state is usually mapped onto only one or a few fixed spectral observables. Introducing an external‐field degree of freedom into q‐BIC platforms is therefore highly desirable for achieving continuous and reversible tunability without changing the device geometry.

To address this requirement, magneto‐optical materials offer a particularly attractive route, as they enable noncontact and reversible spectral control while preserving structural integrity. Compared with structural reconfiguration, magnetic tuning can reshape the optical response without physically altering the resonator geometry. Meanwhile, magneto‐optical q‐BIC systems have advanced rapidly in recent years [34, 35]. Tang et al. showed that an all‐dielectric magneto‐optical metasurface supporting a toroidal‐dipole resonance can strongly enhance Faraday and Kerr rotations [36]. Recent studies have further revealed that magneto‐optical coupling can be used to engineer the polarization topology and chirality of BICs. For example, Zhao et al. demonstrated that magnetic‐field‐induced time‐reversal‐symmetry breaking can lift doubly degenerate BICs and generate spin‐orbit‐locking chiral BICs [37]. Lv et al. reported robust intrinsic C points enabled by magneto‐optical BICs, where magneto‐optical coupling induces Zeeman‐like splitting and circular eigenstates [38]. Tu et al. theoretically demonstrated magnetically tunable BICs with arbitrary polarization covering the full Poincaré sphere, efficient off‐Γ chiral emission, and intrinsic chirality [39]. Zhang et al. reported a BIG‐based magneto‐optical metasurface for high‐Q perfect absorption enabled by q‐BICs [40]. More recent studies have experimentally observed magnetically induced chiral BICs and revealed their switchable far‐field chirality and real‐space topological structures [41, 42]. Together, these studies indicate that q‐BIC platforms and magneto‐optical metasurfaces are individually mature enough to support high‐Q responses, magnetic‐field‐controlled spectral manipulation, and polarization‐topological control. Nevertheless, most existing external‐field tuning strategies in BIC‐based platforms still focus on shifting an isolated resonance, tailoring its radiative leakage, or modifying polarization, chirality, and absorption characteristics, rather than actively reconfiguring the interactions between coupled high‐Q states. In particular, magnetic‐field control over the strong‐coupling regime of two high‐Q resonances remains much less explored. This limitation naturally raises a further question: Can a magnetic field be used not only to tune an isolated q‐BIC resonance but also to reconfigure the strong coupling between two high‐Q resonances and translate such interaction‐level control into reconfigurable refractive‐index sensing?

In this work, we present a magneto‐optical metasurface composed of bismuth iron garnet (BIG) nanoblock dimers, in which a structurally induced q‐BIC couples to a magneto‐structurally enabled q‐BIC, here termed the MS‐BIC. Under an external magnetic field, these two high‐Q resonances are brought into the strong‐coupling regime and hybridize into upper and lower branches (UB and LB), producing a pronounced Rabi splitting in transmission. Multipole decomposition further reveals that the hybridized response is predominantly governed by toroidal dipole (TD) excitations. More importantly, the magnetic field acts not merely as a perturbation for shifting an isolated resonance but as an external degree of freedom for continuously tuning the hybrid‐mode spectrum and coupling strength without altering the device geometry. Numerical refractive‐index sensing results demonstrate high sensitivity in low‐index environments. In addition, magnetic‐field tuning provides multiple addressable resonance observables under a fixed analyte condition, enabling more robust and cross‐validated spectral readout. These results establish a viable route toward magnetically reconfigurable strong‐coupling metasurfaces for refractive‐index sensing and, more broadly, illustrate how an external magnetic field can be used to actively engineer interactions between high‐Q modes for multifunctional nanophotonic operation.

## 2 Structural Design and q‐BIC/MS‐BIC Formation

Figure 1a schematically illustrates the proposed magneto‐optical metasurface, which consists of a periodic array of bismuth iron garnet (BIG) nanoblock dimers supported by a ${\text{SiO}}_{2}$ substrate. The geometry of a single unit cell is shown in Figure 1b,c. The lattice periods are $P_{x}=1000\;nm$ and $P_{y}=500\;nm$. Each BIG nanoblock has an in‐plane width of w=360nm, and the two nanoblocks are arranged along the x direction with an edge‐to‐edge gap of d=140nm. The ${\text{SiO}}_{2}$ substrate thickness is fixed at $d_{2}=100\;nm$. To introduce a controllable in‐plane symmetry breaking, one nanoblock is laterally shifted by Δd along x, and the structural asymmetry is quantified by δ=Δd/d, as shown in Figure 1c. This relative‐shift strategy preserves the compact dimer configuration while directly perturbing the symmetry of the radiative channel, thereby providing a convenient route for converting a symmetry‐protected bound state in the continuum into an observable quasi‐bound state in the continuum [17, 19]. Compared with directly changing the particle size or shape, this approach mainly modifies the radiative accessibility of the mode while keeping the basic dimer framework intact, which is advantageous for tracking the subsequent mode evolution under magnetic perturbation.

**FIGURE 1:**
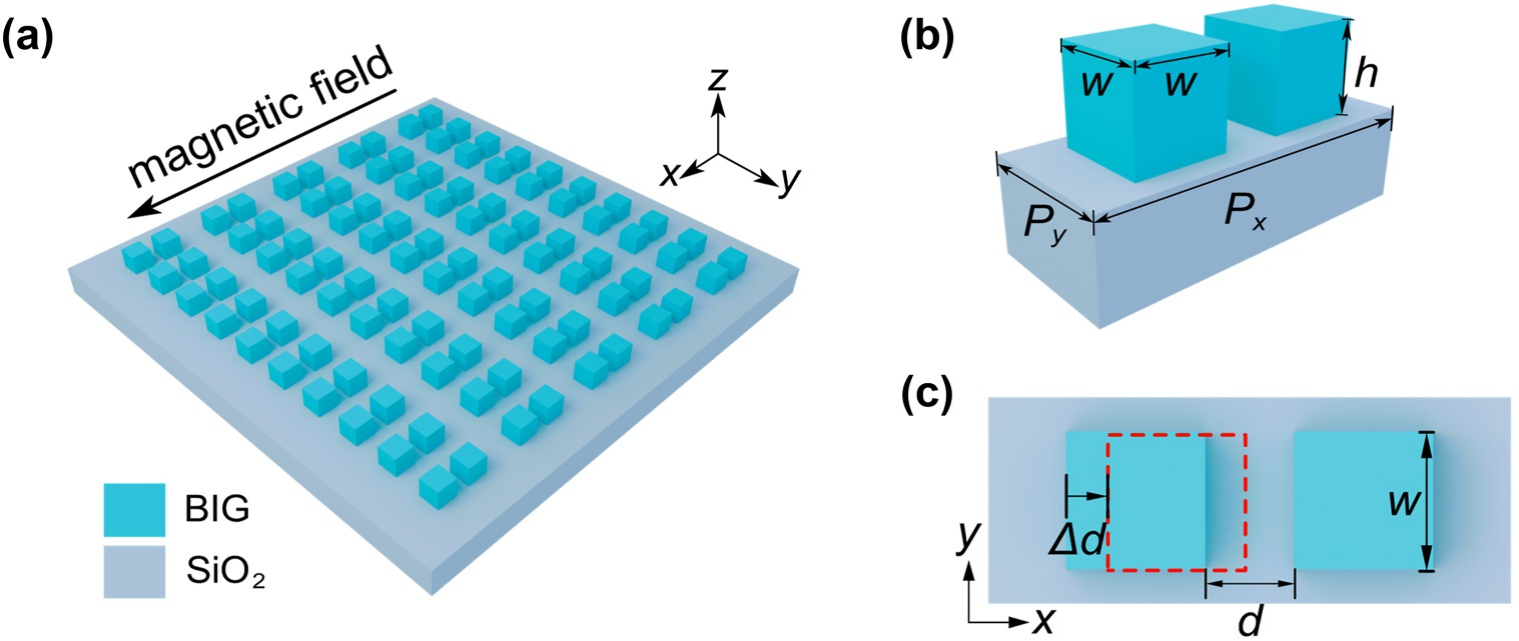
(a) Schematic of the BIG nanoblock dimer array supported by a ${\text{SiO}}_{2}$ substrate under an external static magnetic field applied along the x direction. (b) Three‐dimensional view of the unit cell defining the geometric parameters w, h, $P_{x}$, and $P_{y}$. (c) Top view of the unit cell showing the inter‐block gap d and lateral offset Δd; the structural asymmetry is quantified by δ=Δd/d.

Unless otherwise stated, the working design uses a nanoblock height of h=339nm and a structural asymmetry of δ=0.34. This operating point keeps the resonances clearly observable while retaining their high‐Q characteristics. More specifically, if δ is too small, the radiative coupling of the resonances becomes too weak for robust spectral identification, whereas an excessively large δ would enhance radiation leakage and compromise the high‐Q advantage. The chosen working asymmetry therefore provides a practical balance between spectral observability and linewidth preservation. In the following section, h is scanned over a finite range to tune the spectral detuning between the two modes and to construct the transmission dispersion maps. For modal identification, h=295nm is additionally used below as a representative decoupled condition at which the two resonances are well separated spectrally. This distinction between the working design and the decoupled example is useful for clarifying the subsequent discussion of mode evolution and strong coupling.

The optical response of the metasurface is calculated using the frequency‐domain finite‐element method. Floquet periodic boundary conditions are applied along the lateral directions, and perfectly matched layers are employed along ±z. The structure is excited by a normally incident TM‐polarized plane wave. The refractive index of the ${\text{SiO}}_{2}$ substrate is set to 1.47. Under an external static magnetic field applied along the x direction, BIG exhibits transverse magneto‐optical anisotropy, and its relative permittivity tensor can be written as [34, 35]

(1) $$\varepsilon =\left(\begin{array}{ccc} \varepsilon_{r} & 0 & 0\\ 0 & \varepsilon_{r} & i\varepsilon_{i}\\ 0 & -i\varepsilon_{i} & \varepsilon_{r} \end{array}\right),$$

where $\varepsilon_{r}=6.25$ in the near‐infrared regime and $\varepsilon_{i}$ characterizes the magneto‐optical strength. In the absence of a magnetic field, $\varepsilon_{i}=0$ and the material response is isotropic. Once the magnetic field is applied, time‐reversal symmetry is broken and the off‐diagonal gyrotropic terms become nonzero. In the simulations below, $\varepsilon_{i}$ is used as an effective parameter associated with the applied magnetic field and is varied within $0\leq \varepsilon_{i}\leq 0.1$. Additional simulation details, including the boundary conditions, excitation configuration, and spectral extraction procedure, are provided in Supporting Information S1: Section S1.

The zero‐field case is examined first. As shown in Figure 2a, when $\varepsilon_{i}=0$, the system supports a single ideal bound state, denoted as BIC1, at the symmetric point δ=0. Once δ deviates from zero, the symmetry protection is lifted, allowing the mode to couple to the radiation continuum, and BIC1 evolves into a leaky resonance with a finite linewidth. The corresponding quality‐factor evolution is plotted in Figure 2c. As δ approaches zero, the Q factor rises sharply to the order of $10^{8}$, consistent with the characteristic Q‐factor divergence of a symmetry‐protected BIC. As δ increases, radiation leakage is gradually enhanced and the Q factor decreases continuously. These results confirm that the relative shift Δd is the key geometric parameter governing the transition from an ideal BIC to a radiative q‐BIC state in the present platform.

**FIGURE 2:**
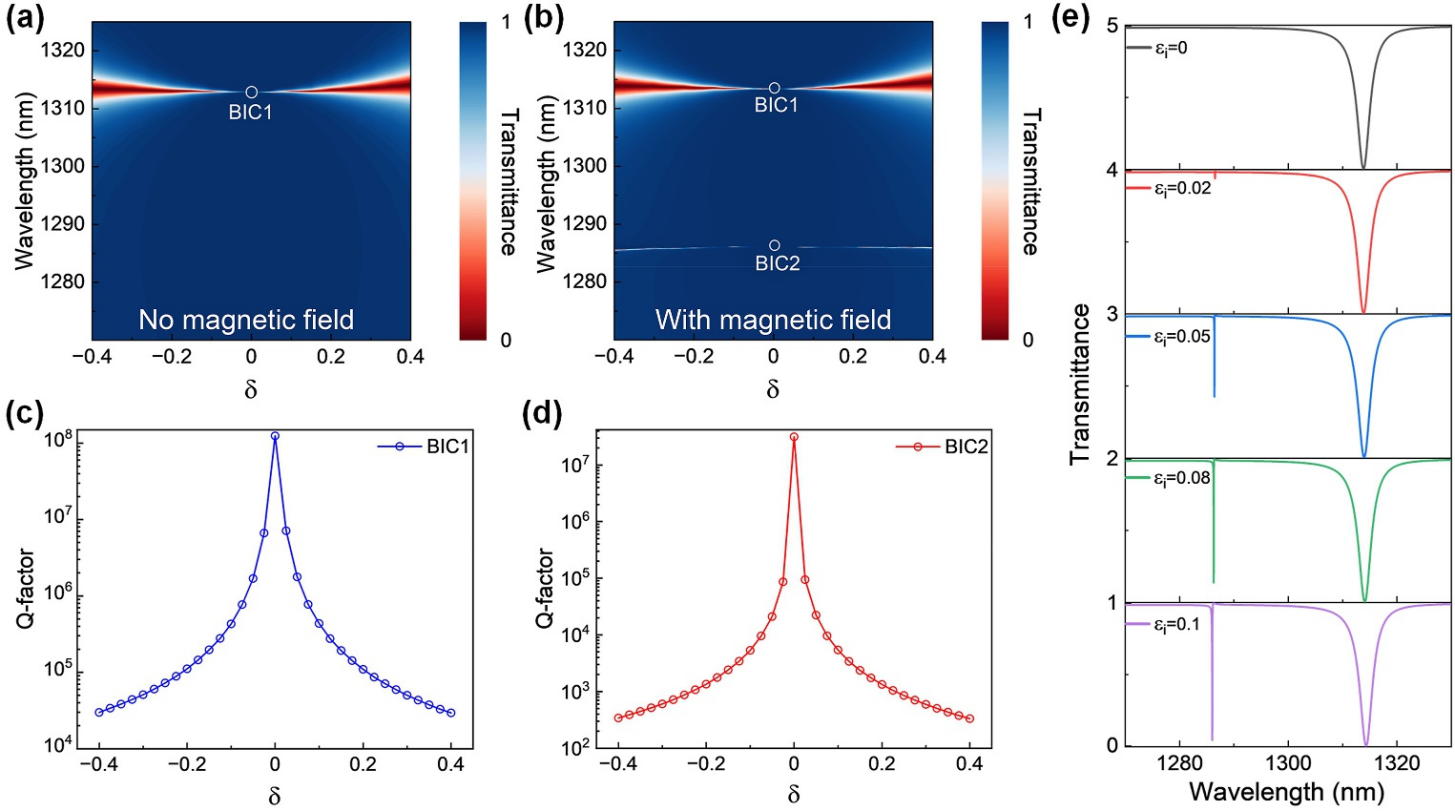
(a) Transmission map as a function of wavelength and structural asymmetry δ in the zero‐field case, showing the BIC1 resonance branch. (b) Transmission map at a finite magneto‐optical strength of $\varepsilon_{i}=0.1$, showing both BIC1 and BIC2; the bright states evolved from them are termed the structural q‐BIC and the MS‐BIC, respectively. (c) Q factor of BIC1 shown in (a) as a function of δ. (d) Q factor of BIC2 shown in (b) as a function of δ. (e) Transmission spectra for selected $\varepsilon_{i}$ values at fixed δ=0.34, showing the spectral evolution from a single resonance to two resonances as the magneto‐optical strength increases.

Once the magnetic field is introduced, a qualitatively different two‐mode response emerges. As shown in Figure 2b, for a representative finite magneto‐optical strength of $\varepsilon_{i}=0.1$, a second ideal bound state, denoted as BIC2, appears in addition to BIC1. This result indicates that the magnetic field preserves the original structural mode while enabling an additional high‐Q branch under the present symmetry configuration. Similar to BIC1, BIC2 is dark at the symmetric point and becomes radiative once a finite asymmetry is introduced. The corresponding Q‐factor evolution in Figure 2d further shows a rapid increase of Q as δ→0, reaching the order of $10^{7}$ near the symmetric limit, which confirms that BIC2 also undergoes a BIC‐to‐q‐BIC transition. In the following, the bright state evolved from BIC1 is referred to as the structural q‐BIC, whereas the bright state evolved from BIC2 is termed the MS‐BIC, emphasizing that its observable form in the working structure requires the combined action of magnetic‐field bias and structural asymmetry. The former modifies the field‐component composition of the MS‐BIC‐related branch, whereas the latter opens the radiative channel required for its spectral observation.

The distinct roles of the magnetic field and structural asymmetry are further clarified by the spectra in Figure 2e, which show how the observable resonances evolve with $\varepsilon_{i}$ at fixed δ=0.34. At $\varepsilon_{i}=0$, only one pronounced resonance dip is present, corresponding to the structural q‐BIC branch. Once the magnetic field is introduced, an additional short‐wavelength dip, corresponding to the MS‐BIC, emerges and becomes increasingly prominent as $\varepsilon_{i}$ increases from 0.02 to 0.1, whereas the structural q‐BIC dip remains clearly visible throughout. This behavior indicates that the magnetic field does not alter the existence of the structural q‐BIC, but instead activates and strengthens an additional resonance branch, namely the MS‐BIC. Accordingly, the roles of the two control parameters are different: δ governs the BIC‐to‐q‐BIC conversion by opening the radiative channel, whereas $\varepsilon_{i}$ mainly controls the emergence and spectral visibility of the MS‐BIC. The two parameters are therefore not redundant, rather, their combined action establishes the observable two‐mode system and provides the physical basis for the strong‐coupling and sensing analyses discussed below.

Accordingly, δ and $\varepsilon_{i}$ play complementary roles in establishing the observable two‐mode system. The external magnetic field modifies the electric‐field component composition of the MS‐BIC‐related branch through magneto‐optically induced $E_{y}$–$E_{z}$ mixing, whereas finite structural asymmetry provides the radiative leakage channel required for the branch to become visible in the far‐field transmission spectrum. As $\varepsilon_{i}$ approaches zero at fixed finite δ, the MS‐BIC‐related branch tends toward a much higher‐Q state with strongly suppressed radiative leakage, and the separate MS‐BIC resonance loses its spectral visibility rather than remaining as an independent bright resonance. In this limit, only the structural q‐BIC remains observable in the transmission spectrum. Thus, the observable MS‐BIC arises from the combined action of magneto‐optical coupling and structural symmetry breaking, as detailed in Supporting Information S1: Figures S1 and S2 and Section S2.

## 3 Strong Coupling and Magnetic Tuning

Having established the formation of the structural q‐BIC and the MS‐BIC, the interaction between them is next examined under finite structural asymmetry and magnetic‐field bias. Unless otherwise stated, the asymmetry parameter is fixed at δ=0.34, such that both resonances remain radiatively accessible while preserving high‐Q characteristics. In this regime, the nanoblock height h is used as the primary geometric parameter to vary the spectral detuning between the two modes, whereas the magneto‐optical strength $\varepsilon_{i}$ provides an external degree of freedom for controlling their interaction. Therefore, h is mainly used to drive the system through the detuning axis, whereas $\varepsilon_{i}$ acts as a field‐controlled tuning parameter for the hybridization strength.

Figure 3a first shows the height‐dependent evolution of the transmission spectra in the absence of a magnetic field. In this case, only a single resonance branch is observed, corresponding to the structural q‐BIC. As the nanoblock height h increases, the resonance wavelength shifts monotonically, reflecting the geometrically governed evolution of this mode. This single‐branch response therefore provides the zero‐field reference for the structural q‐BIC branch discussed below.

**FIGURE 3:**
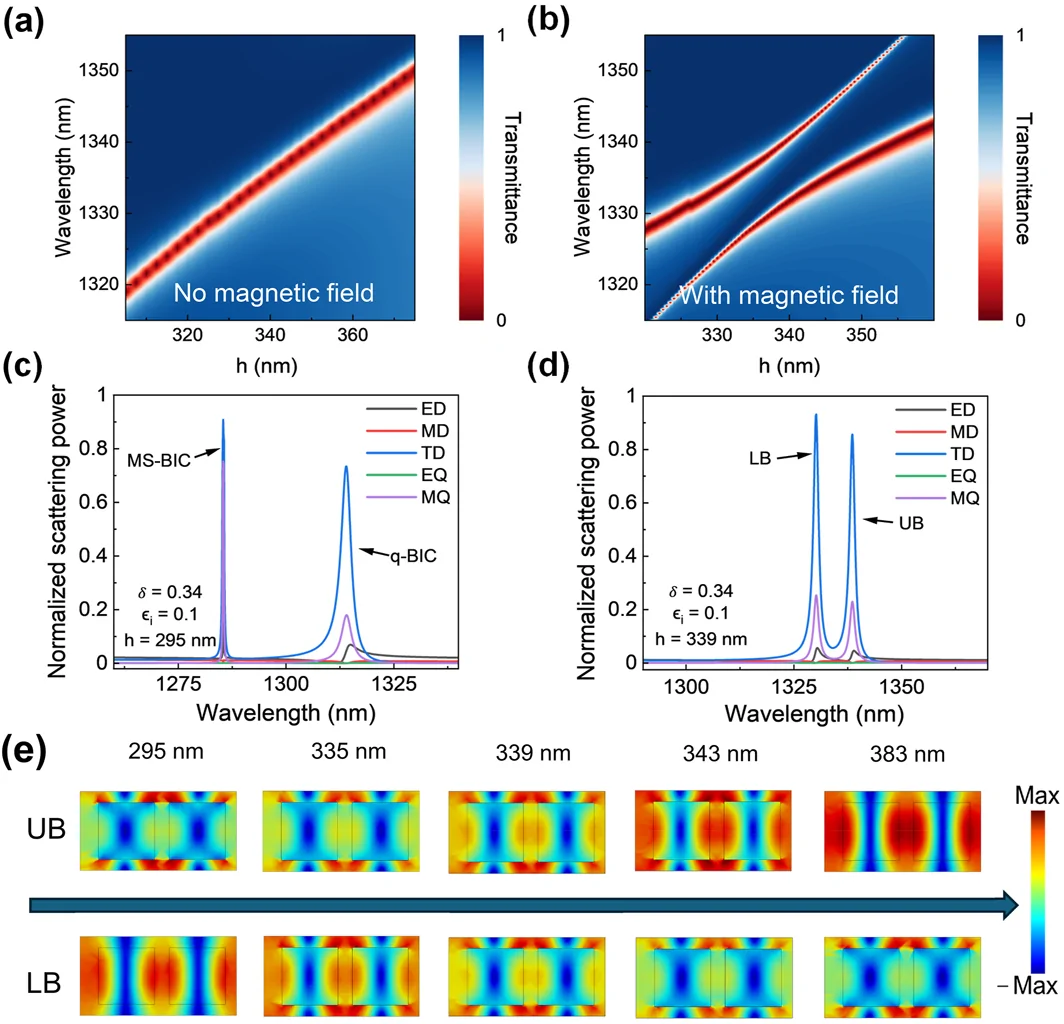
(a) Transmission map as a function of wavelength and nanoblock height h in the absence of magnetic field $\left(\varepsilon_{i}=0\right)$, showing a single structural q‐BIC branch. (b) Transmission map under finite magnetic field $\left(\varepsilon_{i}=0.1\right)$, showing the emergence of an additional MS‐BIC branch and the formation of upper and lower hybrid branches (UB and LB). (c) Multipole decomposition of the scattered power at h=295nm, corresponding to the decoupled regime, where the MS‐BIC and structural q‐BIC are spectrally separated. (d) Multipole decomposition of the scattered power at h=339nm, corresponding to the near‐zero‐detuning strong‐coupling regime. (e) Normalized $E_{z}$ field distributions in the xy plane for UB and LB at selected h values.

When the magnetic field is introduced, the transmission spectra change qualitatively, as shown in Figure 3b. In addition to the structural q‐BIC branch, the MS‐BIC enters the same spectral window and approaches it as h is varied. Near the zero‐detuning region, the two resonances no longer cross, but instead exhibit a pronounced avoided crossing and split into an upper branch (UB) and a lower branch (LB) in the wavelength‐domain dispersion map. This clear anticrossing behavior is the first direct signature of coherent hybridization between the structural q‐BIC and the MS‐BIC, as commonly observed in strongly coupled resonant systems [43, 44, 45, 46, 47]. If the two modes evolved independently, their spectral trajectories would cross once their detuning vanishes. The observed branch repulsion therefore indicates that the relevant eigenstates in this region are no longer the uncoupled parent modes, but hybrid states formed by their mutual interaction.

To further clarify the nature of the two interacting modes, we examine their multipolar responses in the decoupled and hybridized regimes using the Cartesian multipole framework for toroidal excitations [48, 49]. The multipole definitions and normalization procedure are provided in Supporting Information S1: Section S3. As shown in Figure 3c, h=295nm is selected as a representative decoupled condition, under which the two resonances can be treated as effectively uncoupled. Strictly speaking, this does not imply that the coupling is mathematically zero; rather, the spectral separation between the two resonances is sufficiently large that each branch retains a well‐defined dominant modal character and can serve as a practical bare‐mode reference. In this regime, the short‐wavelength resonance, corresponding to the MS‐BIC, is primarily supported by the toroidal dipole (TD), with the magnetic quadrupole (MQ) providing a secondary contribution. By contrast, the long‐wavelength resonance, corresponding to the structural q‐BIC, is nearly purely TD‐like. The electric dipole (ED), magnetic dipole (MD), and electric quadrupole (EQ) contributions remain much weaker over the same spectral range. These results show that both bare modes are TD‐dominated, whereas the MS‐BIC contains a slightly stronger MQ admixture.

At the zero‐detuning point, the multipolar response evolves into that of two hybrid branches. As shown in Figure 3d, at h=339nm both the upper branch (UB) and lower branch (LB) remain predominantly TD‐dominated, whereas the ED, MD, EQ, and MQ contributions are much weaker than the TD contribution. This indicates that the coupling occurs within a common TD‐dominated radiation channel rather than between two modes with completely different radiation characters. The corresponding real‐space field distributions in Figure 3e further support this interpretation. In the decoupled regime, the MS‐BIC and the structural q‐BIC exhibit clearly distinct field profiles, consistent with their different bare‐mode characters. As the system approaches the anticrossing region, the field patterns of the UB and LB show increasingly mixed features, indicating progressive hybridization between the two parent modes. Near the zero‐detuning point, both branches exhibit strongly hybridized field distributions. After passing through the interaction region, the dominant modal characters of the two branches are exchanged. This continuous exchange of modal character is a characteristic signature of avoided‐crossing‐type hybridization and supports the interpretation that the UB and LB are genuine hybrid states rather than two accidentally adjacent resonances.

A quantitative description of the coupling is provided in Figure 4a, where the resonance energies extracted from the transmission spectra are compared with the reference trajectories of the uncoupled structural q‐BIC and MS‐BIC used in the coupled‐oscillator analysis. These reference trajectories represent the bare‐mode continuations of the two parent resonances in the absence of coherent coupling. Because they are reconstructed from the coupled‐oscillator fit, they should be regarded as fitted bare‐mode references rather than directly observed resonances inside the interaction region. In this way, Figure 4a shows both the simulated hybrid branches and the hypothetical spectral evolution that the structural q‐BIC and MS‐BIC would follow without mutual coupling. If the two modes evolved independently, their bare‐mode trajectories would intersect near the zero‐detuning point. Instead, the observed branches repel each other and form a finite minimum separation, directly visualizing the avoided crossing and the onset of hybridization.

**FIGURE 4:**
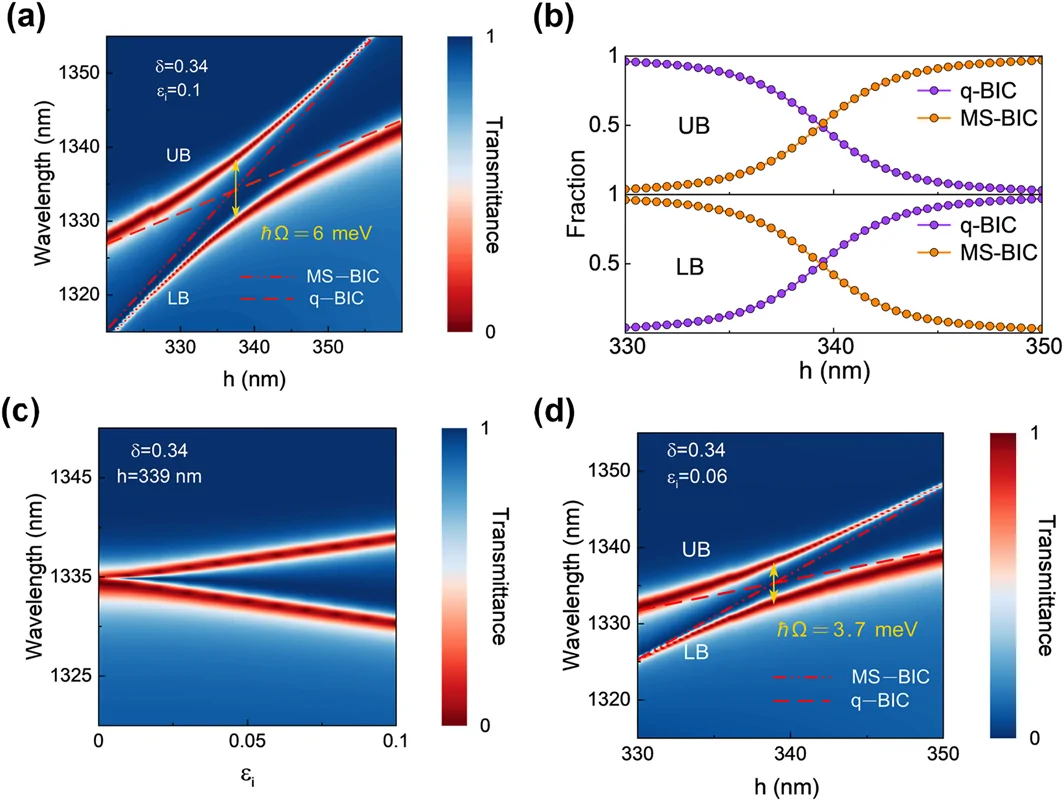
(a) Transmission dispersion versus nanoblock height h, showing the UB and LB hybrid branches and a Rabi‐like splitting of ℏΩ=6meV. (b) Hopfield fractions of the two branches. (c) Transmission dispersion as a function of $\varepsilon_{i}$, revealing magnetic‐field‐induced mode splitting. (d) Strong‐coupling dispersion for a weaker magnetic field, exhibiting a reduced Rabi‐like splitting of ℏΩ=3.7meV.

The splitting is quantified by converting the wavelength‐domain UB and LB resonance positions, $\lambda_{\text{UB}}$ and $\lambda_{\text{LB}}$, into photon energies, $E_{\text{UB}}=hc/\lambda_{\text{UB}}$ and $E_{\text{LB}}=hc/\lambda_{\text{LB}}$, and defining the Rabi‐like splitting as

(2) $$\hbar \Omega =\left|E_{\text{UB}}-E_{\text{LB}}\right|.$$

At the minimum‐separation point, ℏΩ≈6.0meV.

To make the strong‐coupling claim directly verifiable, the linewidths of the uncoupled resonances are also extracted in the energy domain, allowing the splitting and dissipation rates to be compared on the same energy scale. The structural q‐BIC and the MS‐BIC exhibit full widths at half maximum of $\Gamma_{q}=2.2\;meV$ and $\Gamma_{\text{MS}}=1.9\;meV$, respectively. Following the standard linewidth‐based strong‐coupling criterion [43, 44, 45, 47], the condition can be written as

(3) $$\hbar \Omega >\frac{\Gamma_{q}+\Gamma_{\text{MS}}}{2}.$$

For the present system, the observed splitting of ℏΩ≈6.0meV is substantially larger than $\left(\Gamma_{q}+\Gamma_{\text{MS}}\right)/2=2.05\;meV$, confirming that the system operates in the strong‐coupling regime.

The coupled dynamics are further described using a non‐Hermitian coupled‐oscillator model [50]. Additional details on the wavelength‐to‐energy conversion, bare‐mode assignment, Hopfield fractions, and linewidth extraction are provided in Supporting Information S1: Section S4. In the energy‐domain representation, the coupled‐mode equation is written as

(4) $$\left(\begin{array}{cc} E_{q}-i\Gamma_{q}/2 & g\\ g & E_{\text{MS}}-i\Gamma_{\text{MS}}/2 \end{array}\right)\left(\begin{array}{c} \alpha \\ \beta \end{array}\right)=E_{\pm }\left(\begin{array}{c} \alpha \\ \beta \end{array}\right),$$

where $E_{q}$ and $E_{\text{MS}}$ are the resonance energies of the uncoupled structural q‐BIC and MS‐BIC modes, respectively, obtained by converting their extracted resonance wavelengths into photon energies. $\Gamma_{q}$ and $\Gamma_{\text{MS}}$ denote the corresponding full widths at half maximum in energy units, g is the coherent coupling strength, and $E_{\pm }$ are the two hybrid eigenenergies of the coupled system. The coefficients α and β are the Hopfield amplitudes, satisfying $|\alpha {|}^{2}+|\beta {|}^{2}=1$. The UB and LB labels used in the figures follow the upper and lower resonance branches in the wavelength‐domain dispersion maps, rather than being assigned solely according to the + or − signs of the energy eigenvalues. Within this framework, the observed UB and LB branches are naturally interpreted as eigenstates of the coupled two‐mode system rather than as two unrelated resonances.

For physical interpretation, it is useful to introduce the energy detuning as

(5) $$\Delta_{E}=E_{q}-E_{\text{MS}},$$

which quantifies the spectral separation between the two uncoupled parent resonances in the energy domain. In the lossless limit, the hybrid eigenenergies reduce to

(6) $$E_{\pm }=\frac{E_{q}+E_{\text{MS}}}{2}\pm \frac{1}{2}\sqrt{\Delta_{E}^{2}+4g^{2}}.$$

The + and − signs denote the two eigenvalue solutions of the energy‐domain coupled‐oscillator model, following the branch‐labeling convention described in Supporting Information S1: Section S4. This expression shows that the nanoblock height h tunes the coupled system by varying the detuning $\Delta_{E}$ between the two parent modes. As $\Delta_{E}$ approaches zero, the two hybrid eigenenergies exhibit their minimum separation, which is governed by the coupling strength g. Near zero detuning, the fitted coupling strength is g≈3.0meV, giving 2g≈6.0meV, in good agreement with the extracted Rabi‐like splitting of ℏΩ≈6.0meV. This agreement between the spectral splitting and the fitted coupling constant provides additional support for the strong‐coupling interpretation.

The corresponding Hopfield fractions are plotted in Figure 4b, where the UB and LB labels follow the upper and lower branches in the wavelength‐domain dispersion maps. As the nanoblock height h increases, the UB continuously evolves from a structural q‐BIC character to MS‐BIC character, whereas the LB exhibits the opposite trend. For small h, the UB is predominantly structural‐q‐BIC‐like and the LB is mainly MS‐BIC‐like. As the system approaches the anticrossing region, the modal fractions of the two branches gradually converge. At h=339nm, corresponding to the zero‐detuning point, both branches contain nearly equal contributions from the structural q‐BIC and the MS‐BIC, indicating maximally hybridized states. This equal‐weight composition is the Hopfield‐coefficient counterpart of the strongest field mixing observed in Figure 3e. Upon further increasing h, the dominant modal characters of the two branches are exchanged. Such a continuous exchange of modal composition reflects the redistribution of modal weight between the structural q‐BIC and the MS‐BIC under strong coupling, consistent with the field evolution shown in Figure 3e. By tuning h, the hybrid resonance positions and modal composition can therefore be adjusted, enabling the UB and LB resonances to be positioned within a desired spectral window.

An important feature of the present platform is that the strong‐coupling behavior can be actively tuned by the external magnetic field. As shown in Figure 4c, the transmission spectra evolve from a single structural q‐BIC resonance at $\varepsilon_{i}=0$ to a clearly split UB/LB response as $\varepsilon_{i}$ increases at fixed δ=0.34 and h=339nm. With increasing magneto‐optical strength, the UB continuously redshifts whereas the LB blueshifts in the wavelength‐domain spectra, leading to a monotonic increase in their energy‐domain splitting. Throughout this process, the background transmission remains nearly unchanged, suggesting that, within the present model, the magnetic field mainly tunes the coherent interaction between the structural q‐BIC and the MS‐BIC without introducing obvious additional loss. Therefore, the external field mainly reshapes the coupled two‐mode response rather than simply translating a single isolated resonance. This behavior demonstrates that the strong‐coupling state can be externally controlled without modifying the device geometry.

The influence of magnetic‐field strength on the coupling magnitude is further quantified in Figure 4d. For the same structural asymmetry of δ=0.34, reducing the magneto‐optical strength from $\varepsilon_{i}=0.1$ to $\varepsilon_{i}=0.06$ decreases the minimum energy splitting from about ℏΩ=6.0meV to ℏΩ=3.7meV. Although the avoided‐crossing topology is preserved, the reduced minimum separation indicates a weaker coherent interaction under the smaller magnetic field. The external magnetic field therefore serves as an effective tuning knob for the hybrid splitting strength. Although Rabi splittings exceeding 500 meV have recently been reported in molecular, excitonic, and plasmonic strong‐coupling platforms [51, 52, 53], few‐meV splittings have also enabled useful nanophotonic functionalities. For instance, a 5.7‐meV splitting in a BIC–TMDC exciton system has been used for exciton–polariton lifetime engineering [54], an approximately 4‐meV splitting has enabled room‐temperature strong coupling in a BIC‐cavity/single‐photon‐emitter system [55], and vibrational strong coupling with splittings of 1.25–4.45 meV has been explored for molecular spectroscopy, organic‐material sensing, biochemical sensing, and compact mid‐infrared nanophotonic devices [56, 57, 58]. Thus, the present ℏΩ≈6.0meV splitting remains within a functionally relevant range for nanophotonic applications. More importantly, the splitting can be switched on from the zero‐field single‐resonance state and continuously increased up to 6.0 meV by the external magnetic field, which highlights the magnetic reconfigurability of the proposed platform and lays the physical basis for the reconfigurable refractive‐index sensing discussed in the next section.

## 4 Reconfigurable Refractive‐Index Sensing

The magnetically tunable strong‐coupling response established above naturally suggests the proposed metasurface as a platform for reconfigurable refractive‐index sensing. In contrast to conventional passive resonance sensors, where a given analyte state corresponds to a single spectral output, the present system supports two hybrid branches, namely the upper branch (UB) and lower branch (LB), whose spectral positions are jointly determined by the surrounding dielectric environment and the external magnetic field. This dual‐branch response is particularly attractive for sensing because it provides not only two resonance markers within the same device but also an additional degree of freedom for externally reconfiguring the spectral readout without altering the device geometry.

Figure 5a illustrates the sensing concept. The BIG dimer metasurface is integrated into a near‐infrared gas/VOC sensing platform, where the analyte interacts with the resonant surface through a low‐index porous coating layer [59, 60]. In the present design, this coating enhances molecular capture and converts vapor loading into a measurable dielectric perturbation around the metasurface. Such a configuration is well matched to the present high‐Q hybrid modes, whose resonance positions are highly sensitive to small changes in the local dielectric environment sampled by the near field. The coating is modeled as a silica aerogel‐like porous medium with an initial refractive index close to n=1.01. In the sensing simulations, this analyte‐loaded porous coating is modeled as a homogeneous effective‐index layer with a fixed thickness of 2μm, and only its effective refractive index is varied to represent different analyte‐loading conditions. Under analyte exposure, adsorption and capillary condensation inside the porous network increase the local filling fraction and thereby modify the effective refractive index experienced by the hybrid resonances. Accordingly, the quantity n used below does not denote the bulk refractive index of the analyte gas itself, but rather an effective refractive index associated with the analyte‐loaded porous coating and the local near‐field environment of the metasurface. Details of the sensing simulation protocol and the effective‐medium estimate based on the Lorentz–Lorenz relation are provided in Supporting Information S1: Section S5. Such ultralow‐index scaffolds are realistic, as silica aerogel and aerogel‐like mesoporous silica coatings have reported refractive indices approaching that of air [59, 60, 61]. Within this effective‐index picture, the range n=1.01–1.15 therefore captures plausible dielectric variations induced by analyte uptake in low‐index functional overlayers, rather than the intrinsic refractive index range of the analyte gas itself. The response in higher‐index liquid environments, including water and ethanol, is further examined in Supporting Information S1: Section S5, where detectable and magnetically tunable resonance features are obtained, although the liquid‐phase response is less favorable than that in the low‐index porous‐coating regime. Unless otherwise stated, the following sensing results are obtained for a metasurface with δ=0.34 and h=339nm, where the system operates in the strong‐coupling regime and exhibits two well‐resolved hybrid resonances.

**FIGURE 5:**
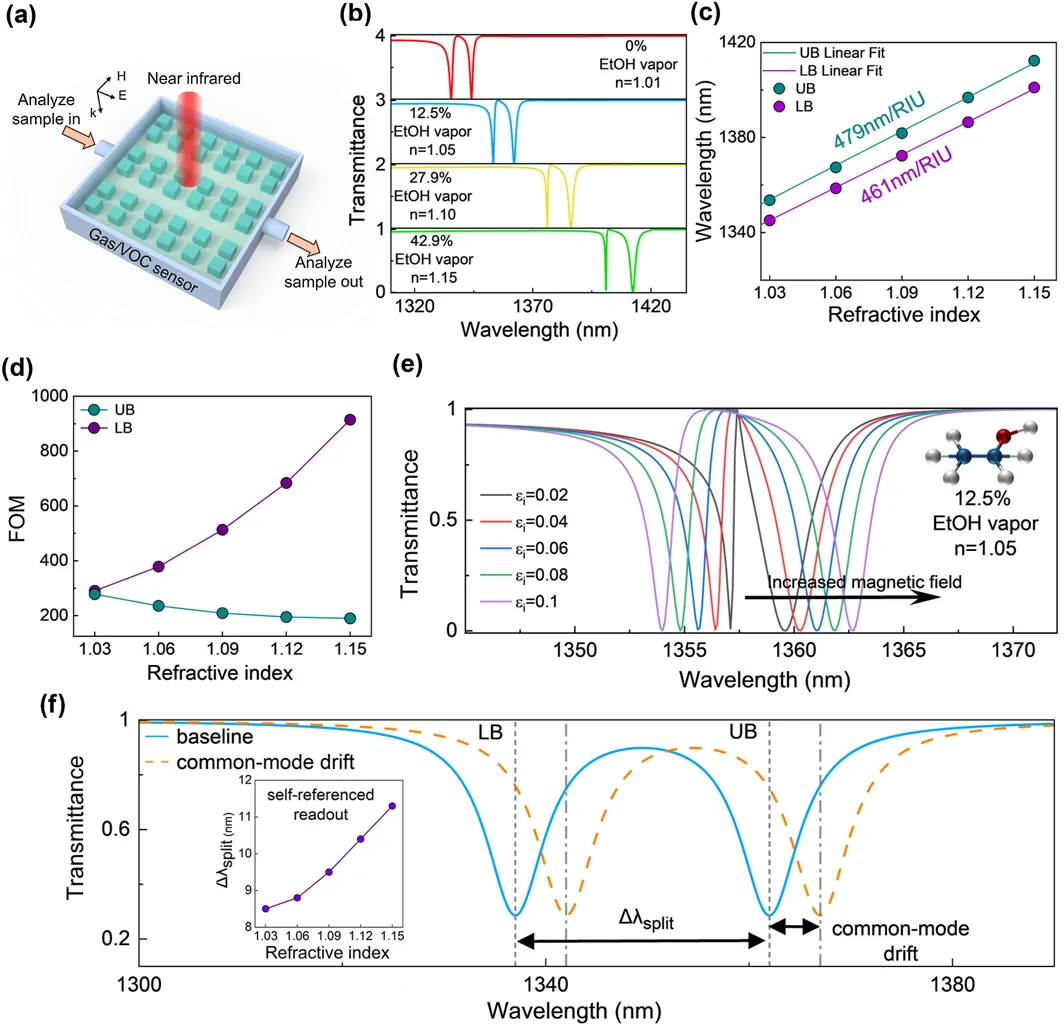
(a) Schematic of the proposed magneto‐optical sensing platform for gas/VOC detection. (b) Transmission spectra for different effective refractive indices n; representative ethanol‐vapor levels are indicated for reference. (c) Resonance wavelengths of the upper branch (UB) and lower branch (LB) as functions of the refractive index, together with linear fits. (d) Figure of merit (FOM) of the UB and LB versus the refractive index. (e) Transmission spectra for different magneto‐optical strengths $\varepsilon_{i}$ under a fixed analyte condition (12.5% EtOH vapor, n=1.05), showing magnetically reconfigured resonance positions. (f) Self‐referenced differential readout based on the absolute UB–LB spectral separation $\Delta \lambda_{\text{split}}=|\lambda_{\text{UB}}-\lambda_{\text{LB}}|$. This differential parameter is less sensitive to common‐mode spectral drift than the absolute resonance positions. The inset shows $\Delta \lambda_{\text{split}}$ as a function of the refractive index.

Figure 5b presents representative transmission spectra for several effective refractive indices. The ethanol‐vapor labels are included as representative analyte conditions, with the percentage denoting the relative vapor pressure at the operating temperature, whereas the optical response is parametrized by the effective refractive index n [60]. In each case, two pronounced resonance dips are observed, corresponding to the UB and LB. As n increases from 1.01 to 1.15, both resonance dips shift monotonically toward longer wavelengths, indicating that the hybrid eigenfrequencies are highly sensitive to dielectric perturbations in the low‐index environment. This redshift is consistent with the general expectation that an increased local dielectric loading lengthens the effective optical path associated with the resonant modes. The nearly linear dependence of both resonance wavelengths on n further enables a straightforward calibration of the sensing response and supports the use of the present system as a readily calibratable refractive‐index sensor.

The sensing performance is quantified using two standard metrics: the refractive‐index sensitivity

(7) $$S=\frac{\Delta \lambda }{\Delta n},$$

and the figure of merit (FOM)

(8) $$FOM=\frac{S}{\Delta \lambda_{\text{FWHM}}},$$

where Δλ is the resonance shift induced by an effective refractive‐index change Δn, and $\Delta \lambda_{\text{FWHM}}$ is the full width at half maximum of the corresponding resonance dip. The extracted resonance wavelengths of the UB and LB are plotted in Figure 5c, together with linear fits. The corresponding sensitivities are approximately 479nm/RIU for the UB and 461nm/RIU for the LB. These values indicate that both hybrid branches are comparably responsive to refractive‐index perturbations, which is consistent with the fact that both resonances originate from the same strongly coupled two‐mode system and probe essentially the same analyte‐modified near‐field environment. The resulting FOM values are shown in Figure 5d. Owing to the narrow linewidth preserved by the hybrid high‐Q states, the LB achieves a maximum FOM of about $915\;{RIU}^{-1}$ at n=1.15, whereas the UB maintains a lower FOM and exhibits a gradual decrease across the same refractive‐index range. The higher FOM of the LB mainly originates from its narrower linewidth under the present operating conditions, even though the UB and LB exhibit comparable refractive‐index sensitivities. This trend indicates that, in the present working window, the sensing performance is determined not only by how strongly the resonance wavelength shifts but also by how sharply each hybrid branch remains spectrally resolved. In this sense, the LB provides the more favorable sensing feature when a narrow linewidth and high readout precision are prioritized, whereas the UB still serves as an additional independent spectral observable with comparable sensitivity.

To provide a clearer performance context, Table 1 compares the present device with representative recent BIC‐based refractive‐index sensors, including passive, electrically tunable, electro‐optically tunable, and phase‐change‐tunable platforms. Because these sensors differ in material system, spectral range, and sensing configuration, the comparison is intended as a representative benchmark rather than a direct ranking. The proposed sensor exhibits competitive sensitivity and FOM while introducing magnetic‐field control as an additional reconfiguration degree of freedom.

**TABLE 1:** Comparison of the proposed sensor with representative BIC‐based refractive‐index sensors.

Work	System	BIC/sensing mode	Operation method	Sensitivity	FOM
Hsiao et al. [62]	Si nanobar‐pair metasurface	TD–EQ q‐BIC	Passive	608 nm ${\text{RIU}}^{-1}$	46 ${\text{RIU}}^{-1}$
Watanabe and Iwanaga [30]	Si nanogap metasurface	Nanogap‐enhanced q‐BIC	Passive	317 nm ${\text{RIU}}^{-1}$	Up to 239 ${\text{RIU}}^{-1}$
Li et al. [33]	Amorphous‐Si metasurface	MTD q‐BIC	Passive	413 nm ${\text{RIU}}^{-1}$	66 ${\text{RIU}}^{-1}$
Liu et al. [63]	Graphene–${\text{TiO}}_{2}$ grating	Phase‐difference‐induced q‐BIC	Electrical tuning	810 nm ${\text{RIU}}^{-1}$	$5.7\times 10^{4}$ ${\text{RIU}}^{-1}$
Tang et al. [64]	${\text{LiNbO}}_{3}$ chiral metasurface	Chiral BIC	Electro‐optic tuning	590 nm ${\text{RIU}}^{-1}$	$5.9\times 10^{4}$ ${\text{RIU}}^{-1}$
Wang et al. [10]	GST225 metasurface	Multi‐band quasi‐BIC	Phase‐change tuning	1017 nm ${\text{RIU}}^{-1}$	N.R.
This work	BIG magneto‐optical dimer metasurface	Coupled structural q‐BIC/MS‐BIC	Magnetic‐field tuning	**479/461 nm** ${\text{RIU}}^{-1}$	**915** ${\text{RIU}}^{-1}$

*Note:* The bold values indicate the sensing performance of this work: 479 and 461 nm RIU−1 are the UB and LB sensitivities from Figure 5c, respectively, and 915 RIU−1 is the maximum FOM from Figure 5d.

Abbreviation: N.R., not reported.

A distinctive feature of the present sensor is that its spectral readout can be actively reconfigured by the magnetic field without changing either the device geometry or the analyte condition. This functionality is illustrated in Figure 5e, where the transmission spectra are plotted for a fixed sensing state corresponding to 12.5% ethanol vapor and an effective refractive index of n=1.05, whereas the magneto‐optical strength is varied from $\varepsilon_{i}=0.02$ to 0.1. Under this fixed analyte condition, the UB and LB do not remain at fixed wavelengths; instead, they shift continuously in opposite directions as the magnetic field increases, leading to an enlarged spectral separation between the two resonance dips. Therefore, a single analyte‐loaded porous environment can be interrogated under multiple magnetically addressable spectral states. The value of this functionality lies not only in providing more resonance features but also in enabling the same sensing state to be read out through externally selected spectral configurations. Such reconfigurable readout provides redundant observables for cross‐validation, can reduce the influence of common‐mode spectral drift, and offers an additional constraint for resonance fitting. It also extends the practical operating window of the device by allowing the readout wavelength to be selected through external magnetic control. In this sense, the magnetic field acts as a reconfiguration knob for the dual‐branch sensing response itself rather than as a secondary perturbation applied to a fixed spectral response.

In addition to the absolute resonance positions, the analyte state can also be characterized by the spectral separation between the two hybrid branches. As illustrated in Figure 5f, this separation can be defined as a differential readout parameter,

(9) $$\Delta \lambda_{\text{split}}=|\lambda_{\text{UB}}-\lambda_{\text{LB}}|.$$

$\lambda_{\text{UB}}$ and $\lambda_{\text{LB}}$ denote the resonance wavelengths of the upper and lower branches, respectively, and the absolute value indicates that $\Delta \lambda_{\text{split}}$ represents the spectral separation between the two hybrid resonances. Compared with conventional absolute‐peak readout, $\Delta \lambda_{\text{split}}$ provides an additional relative observable extracted from the same pair of hybrid resonances. In a realistic measurement, slow common‐mode spectral drifts, such as those caused by illumination‐source instability or spectrometer calibration offsets, may shift both resonance wavelengths in a similar direction. In contrast, the relative separation between the UB and LB is less sensitive to such common‐mode shifts, as schematically indicated in Figure 5f. The inset further shows that $\Delta \lambda_{\text{split}}$ varies systematically with the effective refractive index, confirming its potential as a self‐referenced auxiliary sensing parameter. Therefore, the proposed sensor can support both absolute‐peak readout based on the individual UB and LB positions and differential readout based on their spectral separation. This coexistence of absolute and relative sensing channels highlights the advantage of the magnetically reconfigurable strong‐coupling platform, offering a more flexible and potentially more robust sensing strategy than conventional single‐resonance passive sensors.

It should be noted that, in practical porous‐coating‐based sensing, humidity may act as an important interferent because water vapor can also be adsorbed into the porous network and modify the effective refractive index of the coating. This humidity‐induced refractive‐index variation may cause a background spectral shift that overlaps with the response of the target analyte. To mitigate this effect, the dry‐state optical response of the porous coating can be measured in advance and used as a reference baseline. The spectra obtained under humid or analyte‐containing environments can then be calibrated relative to this reference, so that humidity‐induced background contributions can be partially compensated. Neither the magnetic configuration nor the differential splitting readout $\Delta \lambda_{\text{split}}$ is intended to provide direct chemical specificity between ethanol vapor and water vapor by itself. Such distinction would require suitable functional coatings or additional humidity‐calibration procedures.

## 5 Conclusion

In summary, we have presented a magneto‐optical metasurface in which an external magnetic field serves not merely as a means of shifting an isolated resonance but as an effective degree of freedom for reconfiguring the interaction between two high‐Q modes without altering the device geometry. By combining structural symmetry breaking with magneto‐optical anisotropy in a BIG dimer array, strong coupling is established between a structural q‐BIC and a magneto‐structurally induced q‐BIC (MS‐BIC), giving rise to upper and lower hybrid branches with a toroidal‐dipole‐dominated response and a magnetically tunable avoided crossing. Within the present design window, the hybrid splitting can be continuously tuned from 3.7 to 6.0meV. Benefiting from the high‐Q nature of the coupled states, the proposed platform further enables dual‐branch refractive‐index sensing in low‐index environments, with sensitivities of approximately 479 and 461nm/RIU for the upper and lower branches, respectively, and a maximum figure of merit of $915\;{RIU}^{-1}$. More importantly, magnetic tuning provides multiple addressable spectral responses under a fixed analyte condition, allowing the sensing state to be inferred not only from the absolute positions of the UB and LB, but also from their spectral separation. This additional differential observable offers a self‐referenced readout channel that can help mitigate the influence of common‐mode spectral drift and provide an auxiliary constraint for resonance fitting. These results establish magnetically controlled strong‐coupling metasurfaces as a promising platform for reconfigurable refractive‐index sensing and highlight the broader potential of external‐field control for engineering interactions between high‐Q modes in multifunctional nanophotonic devices.

## Author Contributions

**Runmin Liu:** investigation, methodology, writing – original draft. **Shengyi Wang:** writing – original draft, validation, formal analysis. **Liangui Deng:** methodology, formal analysis, funding acquisition. **Hua Ge:** visualization, formal analysis. **Qiu Wang:** project administration, funding acquisition, writing – review and editing. **Bowen Jia:** funding acquisition, supervision, writing – review and editing, conceptualization.

## Funding

National Natural Science Foundation of China (Grants 62104174, 62205253, 62405225), Natural Science Foundation of Wuhan (Grant 2026040101030002), and Hubei Provincial Key Research and Development Program of China (Grant 2024BAA003).

## Conflicts of Interest

The authors declare no conflicts of interest.

## Supporting information


Supporting Information

## Data Availability

Data underlying the results presented in this article are not publicly available but may be obtained from the authors upon reasonable request.

## References

[1] M. Wuttig , H. Bhaskaran , and T. Taubner , “Phase‐Change Materials for Non‐Volatile Photonic Applications,” Nature Photonics 11, no. 8 (2017): 465–476, 10.1038/nphoton.2017.126.

[2] A. M. Shaltout , V. M. Shalaev , and M. L. Brongersma , “Spatiotemporal Light Control With Active Metasurfaces,” Science 364, no. 6441 (2019): eaat3100, 10.1126/science.aat3100. 31097638

[3] J. Sautter , I. Staude , M. Decker , et al., “Active Tuning of All‐Dielectric Metasurfaces,” ACS Nano 9, no. 4 (2015): 4308–4315, 10.1021/acsnano.5b00723. 25748581

[4] A. Komar , R. Paniagua‐Domínguez , A. Miroshnichenko , et al., “Dynamic Beam Switching by Liquid Crystal Tunable Dielectric Metasurfaces,” ACS Photonics 5, no. 5 (2018): 1742–1748, 10.1021/acsphotonics.7b01343.

[5] Y. Zhao , Z. Liu , C. Li , et al., “Mechanically Reconfigurable Metasurfaces: Fabrications and Applications,” npj Nanophotonics 1, no. 1 (2024): 16, 10.1038/s44310-024-00010-z.

[6] M. Cotrufo , S. B. Sulejman , L. Wesemann , et al., “Reconfigurable Image Processing Metasurfaces With Phase‐Change Materials,” Nature Communications 15, no. 1 (2024): 4483, 10.1038/s41467-024-48783-3. PMC11130277 38802353

[7] J. Sisler , P. Thureja , M. Y. Grajower , R. Sokhoyan , I. Huang , and H. A. Atwater , “Electrically Tunable Space–Time Metasurfaces at Optical Frequencies,” Nature Nanotechnology 19, no. 10 (2024): 1491–1498, 10.1038/s41565-024-01728-9. 39048705

[8] Z. Ma , T. Tian , Y. Liao , et al., “Electrically Switchable 2N‐Channel Wave‐Front Control for Certain Functionalities With N Cascaded Polarization‐Dependent Metasurfaces,” Nature Communications 15, no. 1 (2024): 8370, 10.1038/s41467-024-52676-w. PMC11436973 39333169

[9] J. Siegel , S. Kim , M. Fortman , et al., “Electrostatic Steering of Thermal Emission With Active Metasurface Control of Delocalized Modes,” Nature Communications 15, no. 1 (2024): 3376, 10.1038/s41467-024-47229-0. PMC11032313 38643246

[10] S. Y. Wang , Q. Wang , H. Luo , et al., “Dynamically Tunable Multi‐Band Quasi‐Permittivity‐Asymmetric BIC in the GST225 Metasurface,” Optics Letters 49, no. 17 (2024): 4767, 10.1364/ol.531446. 39207959

[11] Y. Wei , L. Si , L. Dong , et al., “A mid‐IR Tunable Graphene Metasurface for Ultrasensitive Molecular Fingerprint Retrieval and Refractive Index Sensing,” Journal of Materials Chemistry C 11, no. 47 (2023): 16501–16508, 10.1039/d3tc03554a.

[12] D.‐C. Marinica , A. G. Borisov , and S. V. Shabanov , “Bound States in the Continuum in Photonics,” Physical Review Letters 100, no. 18 (2008): 183902, 10.1103/physrevlett.100.183902. 18518374

[13] C. W. Hsu , B. Zhen , A. D. Stone , J. D. Joannopoulos , and M. Soljačić , “Bound States in the Continuum,” Nature Reviews Materials 1, no. 9 (2016): 16048, 10.1038/natrevmats.2016.48.

[14] B. Zhen , C. W. Hsu , L. Lu , A. D. Stone , and M. Soljačić , “Topological Nature of Optical Bound States in the Continuum,” Physical Review Letters 113, no. 25 (2014): 257401, 10.1103/physrevlett.113.257401. 25554906

[15] G. Xu , H. Xing , Z. Xue , et al., “Recent Advances and Perspective of Photonic Bound States in the Continuum,” Ultrafast Science 3 (2023): 0033, 10.34133/ultrafastscience.0033.

[16] M. Kang , T. Liu , C. T. Chan , and M. Xiao , “Applications of Bound States in the Continuum in Photonics,” Nature Reviews Physics 5, no. 11 (2023): 659–678, 10.1038/s42254-023-00642-8.

[17] K. Koshelev , S. Lepeshov , M. Liu , A. Bogdanov , and Y. Kivshar , “Asymmetric Metasurfaces With High‐Q Resonances Governed by Bound States in the Continuum,” Physical Review Letters 121, no. 19 (2018): 193903, 10.1103/physrevlett.121.193903. 30468599

[18] M. V. Rybin , K. L. Koshelev , Z. F. Sadrieva , et al., “High‐Q Supercavity Modes in Subwavelength Dielectric Resonators,” Physical Review Letters 119, no. 24 (2017): 243901, 10.1103/physrevlett.119.243901. 29286713

[19] T. Sang , Q. Mi , C. Yang , et al., “Achieving Asymmetry Parameter‐Insensitive Resonant Modes Through Relative Shift–Induced Quasi‐Bound States in the Continuum,” Nanophotonics 13, no. 8 (2024): 1369–1377, 10.1515/nanoph-2023-0673. PMC11636510 39679228

[20] S. Jahani and Z. Jacob , “All‐Dielectric Metamaterials,” Nature Nanotechnology 11, no. 1 (2016): 23–36, 10.1038/nnano.2015.304. 26740041

[21] S. Kruk and Y. Kivshar , “Functional Meta‐Optics and Nanophotonics Governed by Mie Resonances,” ACS Photonics 4, no. 11 (2017): 2638–2649, 10.1021/acsphotonics.7b01038.

[22] Y. Liu , Q. Zhang , X. Xing , et al., “Terahertz Narrowband Filter Metasurfaces Based on Bound States in the Continuum,” Optics Express 31, no. 21 (2023): 35272, 10.1364/oe.503399. 37859262

[23] P. Ren , Z. Huang , S. Luo , et al., “Quasi‐BICs Enhanced Second Harmonic Generation From WSe _2_ Monolayer,” Nanophotonics 13, no. 18 (2024): 3449–3456, 10.1515/nanoph-2024-0108. PMC11501586 39634849

[24] A. Kodigala , T. Lepetit , Q. Gu , B. Bahari , Y. Fainman , and B. Kanté , “Lasing Action From Photonic Bound States in Continuum,” Nature 541, no. 7636 (2017): 196–199, 10.1038/nature20799. 28079064

[25] Y. Ren , P. Li , Z. Liu , et al., “Low‐Threshold Nanolasers Based on Miniaturized Bound States in the Continuum,” Science Advances 8, no. 51 (2022): eade8817, 10.1126/sciadv.ade8817. PMC9788758 36563161

[26] K. Sun , Y. Cai , L. Huang , and Z. Han , “Ultra‐Narrowband and Rainbow‐free Mid‐infrared Thermal Emitters Enabled by a Flat Band Design in Distorted Photonic Lattices,” Nature Communications 15, no. 1 (2024): 4019, 10.1038/s41467-024-48499-4. PMC11091152 38740756

[27] F. Yesilkoy , E. R. Arvelo , Y. Jahani , et al., “Ultrasensitive Hyperspectral Imaging and Biodetection Enabled by Dielectric Metasurfaces,” Nature Photonics 13, no. 6 (2019): 390–396, 10.1038/s41566-019-0394-6.

[28] A. Tittl , A. Leitis , M. Liu , et al., “Imaging‐Based Molecular Barcoding With Pixelated Dielectric Metasurfaces,” Science 360, no. 6393 (2018): 1105–1109, 10.1126/science.aas9768. 29880685

[29] A. Leitis , A. Tittl , M. Liu , et al., “Angle‐Multiplexed All‐Dielectric Metasurfaces for Broadband Molecular Fingerprint Retrieval,” Science Advances 5, no. 5 (2019): eaaw2871, 10.1126/sciadv.aaw2871. PMC6527437 31123705

[30] K. Watanabe and M. Iwanaga , “Nanogap Enhancement of the Refractometric Sensitivity at Quasi‐Bound States in the Continuum in All‐Dielectric Metasurfaces,” Nanophotonics 12, no. 1 (2023): 99–109, 10.1515/nanoph-2022-0565. PMC11501443 39633629

[31] S. Romano , G. Zito , S. Torino , et al., “Label‐Free Sensing of Ultralow‐Weight Molecules With All‐Dielectric Metasurfaces Supporting Bound States in the Continuum,” Photonics Research 6, no. 7 (2018): 726, 10.1364/prj.6.000726.

[32] T. van Loon , M. Liang , T. Delplace , et al., “Refractive Index Sensing Using Quasi‐Bound States in the Continuum in Silicon Metasurfaces,” Optics Express 32, no. 8 (2024): 14289, 10.1364/oe.514787. 38859379

[33] N. Li , H. Chen , Y. Zhao , et al., “Ultrasensitive Metasurface Sensor Based on Quasi‐Bound States in the Continuum,” Nanophotonics 14, no. 4 (2025): 485–494, 10.1515/nanoph-2024-0728. PMC11833742 39975632

[34] E. Yao , Z. Su , Y. Bi , Y. Wang , and L. Huang , “Tunable Quasi‐Bound States in the Continuum in Magneto‐Optical Metasurfaces,” Journal of Physics D: Applied Physics 57, no. 37 (2024): 375104, 10.1088/1361-6463/ad5215.

[35] J. Li , Z. Wu , D. Zhang , Y. Sun , W. Liu , and T. Yu , “Nonreciprocal Toroidal Dipole Resonance and One‐Way Quasi‐Bound State in the Continuum,” Optics Letters 49, no. 5 (2024): 1313, 10.1364/ol.516427. 38427001

[36] Q. Tang , D. Zhang , T. Liu , et al., “Enhancing Faraday and Kerr Rotations Based on the Toroidal Dipole Mode in an All‐Dielectric Magneto‐Optical Metasurface,” Optics Letters 48, no. 13 (2023): 3451, 10.1364/ol.492913. 37390153

[37] X. Zhao , J. Wang , W. Liu , et al., “Spin‐Orbit‐Locking Chiral Bound States in the Continuum,” Physical Review Letters 133, no. 3 (2024): 036201, 10.1103/physrevlett.133.036201. 39094160

[38] W. Lv , H. Qin , Z. Su , et al., “Robust Generation of Intrinsic C Points With Magneto‐Optical Bound States in the Continuum,” Science Advances 10, no. 46 (2024): eads0157, 10.1126/sciadv.ads0157. PMC11566988 39546605

[39] Q.‐A. Tu , H. Zhou , D. Zhao , Y. Meng , M. Gong , and Z. Gao , “Magnetically Tunable Bound States in the Continuum With Arbitrary Polarization and Intrinsic Chirality,” Photonics Research 12, no. 12 (2024): 2972, 10.1364/prj.539830.

[40] H. Zhang , G. P. Wang , and K. Wu , “Magneto‐Optical Metasurfaces for High‐Q Perfect Absorption With Quasi‐Bound States in the Continuum,” Optics Letters 49, no. 16 (2024): 4654, 10.1364/OL.536497. 39146127

[41] M. Gong , Q. Zhen , Y. Tang , et al., “Observation of Chiral Bound States in the Continuum in Self‐Biased Magneto‐Optical Photonic Crystals,” Proceedings of the National Academy of Sciences 123, no. 21 (2026): e2536341123, 10.1073/pnas.2536341123. PMC13214002 42166237

[42] X. Zhao , J. Chen , J. Wang , et al., arXiv Preprint arXiv:2602.22634, (2026).

[43] P. Törmä and W. L. Barnes , “Strong Coupling Between Surface Plasmon Polaritons and Emitters: A Review,” Reports on Progress in Physics 78, no. 1 (2015): 013901, 10.1088/0034-4885/78/1/013901. 25536670

[44] D. G. Baranov , M. Wersäll , J. Cuadra , T. J. Antosiewicz , and T. Shegai , “Novel Nanostructures and Materials for Strong Light–Matter Interactions,” ACS Photonics 5, no. 1 (2018): 24–42, 10.1021/acsphotonics.7b00674.

[45] Y. Chen , W. Chen , X. Kong , D. Wu , J. Chu , and C.‐W. Qiu , “Can Weak Chirality Induce Strong Coupling Between Resonant States?,” Physical Review Letters 128, no. 14 (2022): 146102, 10.1103/physrevlett.128.146102. 35476494

[46] S. Y. Wang , W. Y. Li , H. F. Kang , et al., “Giant Strong Coupling in a Q‐BICs’ Tetramer Metasurface,” Optics Letters 49, no. 15 (2024): 4154, 10.1364/ol.533463. 39090882

[47] M. Hertzog , M. Wang , J. Mony , and K. Börjesson , “Strong Light–Matter Interactions: A New Direction Within Chemistry,” Chemical Society Reviews 48, no. 3 (2019): 937–961, 10.1039/c8cs00193f. PMC6365945 30662987

[48] T. Kaelberer , V. A. Fedotov , N. Papasimakis , D. P. Tsai , and N. I. Zheludev , “Toroidal Dipolar Response in a Metamaterial,” Science 330, no. 6010 (2010): 1510–1512, 10.1126/science.1197172. 21051597

[49] V. Savinov , V. A. Fedotov , and N. I. Zheludev , “Toroidal Dipolar Excitation and Macroscopic Electromagnetic Properties of Metamaterials,” Physical Review B 89, no. 20 (2014): 205112, 10.1103/physrevb.89.205112.

[50] L. Yue , P. Xie , S. Shen , Q. Ding , and W. Wang , “Chiral Effects in Strongly Coupled Resonant Metasurfaces With Epsilon‐Near‐Zero Guided Modes,” Physical Review B 112, no. 3 (2025): 035408, 10.1103/kjqf-szyp.

[51] L. Zhang , K. Ridier , O. Y. Horniichuk , et al., “Reversible Switching of Strong Light–Matter Coupling Using Spin‐Crossover Molecular Materials,” Journal of Physical Chemistry Letters 14, no. 30 (2023): 6840–6849, 10.1021/acs.jpclett.3c01136. 37487224

[52] S. B. Anantharaman , J. Lynch , M. Aleksich , et al., “Ultrastrong Light–Matter Coupling in Two‐Dimensional Metal–Organic Chalcogenolates,” Nature Photonics 19, no. 3 (2025): 322–328, 10.1038/s41566-024-01590-0.

[53] B. B. Shurtleff , R.‐M. Friedrich , T. Strunskus , M. Elbahri , and F. Faupel , “Hybrid Polaritonic Switch With Light‐Controlled Rabi Splitting in Molecular Plasmonic System,” Nanoscale Advances 8, no. 10 (2026): 3248–3255, 10.1039/d5na01061f. PMC13126553 42063410

[54] K. L. Koshelev , S. K. Sychev , Z. F. Sadrieva , A. A. Bogdanov , and I. V. Iorsh , “Strong Coupling Between Excitons in Transition Metal Dichalcogenides and Optical Bound States in the Continuum,” Physical Review B 98, no. 16 (2018): 161113, 10.1103/physrevb.98.161113.

[55] T. T. H. Do , M. Nonahal , C. Li , et al., “Room‐Temperature Strong Coupling in a Single‐Photon Emitter‐Metasurface System,” Nature Communications 15, no. 1 (2024): 2281, 10.1038/s41467-024-46544-w. PMC10937668 38480721

[56] W. Sun , F. Yang , X. Shi , et al., “All‐Dielectric quasi‐BIC Metasurface With Strong Coupling Control by Adjusting the Loss—Potential for Vibrational Strong Coupling,” Applied Physics Letters 126, no. 26 (2025): 261703, 10.1063/5.0276130.

[57] Y. Liu , E. Maqbool , and Z. Han , “The Strong Coupling Effect Between Metallic Split‐Ring Resonators and Molecular Vibrations in Polymethyl Methacrylate,” Sensors 24, no. 8 (2024): 2479, 10.3390/s24082479. PMC11055064 38676096

[58] P. Xie and Y. Cheng , “Manipulating Coherent Interaction of Molecular Vibrations With Quasibound States in the Continuum in All‐Dielectric Metasurfaces,” Physical Review B 108, no. 15 (2023): 155412, 10.1103/physrevb.108.155412.

[59] M. Modaresialam , G. Bordelet , Z. Chehadi , et al., “Enhanced Refractive Index Sensitivity Through Combining a Sol–Gel Adsorbate With a TiO2 Nanoimprinted Metasurface for Gas Sensing,” ACS Applied Materials & Interfaces 13, no. 44 (2021): 53021, 10.1021/acsami.1c13248. 34708655

[60] O. Dalstein , M. Tabo , E. Alvarez , et al., “Method to Detect Ethanol Vapor in High Humidity by Direct Reflection on a Xerogel Coating,” ACS Applied Materials & Interfaces 11, no. 4 (2019): 4439, https://pubs.acs.org/doi/abs/10.1021/acsami.8b20479. 10.1021/acsami.8b20479 30629407

[61] J. Wang , D. Petit , and S. Ren , “Transparent Thermal Insulation Silica Aerogels,” Nanoscale Advances 2, no. 12 (2020): 5504–5515, 10.1039/d0na00655f. PMC9417477 36133881

[62] H.‐H. Hsiao , Y.‐C. Hsu , A.‐Y. Liu , J.‐C. Hsieh , and Y.‐H. Lin , “Ultrasensitive Refractive Index Sensing Based on the Quasi‐Bound States in the Continuum of All‐Dielectric Metasurfaces,” Advanced Optical Materials 10, no. 19 (2022): 2200812, 10.1002/adom.202200812.

[63] W. Liu , B. Yan , and X. Jiang , “High Performance Sensor Based on Phase Difference Induced Quasi‐BIC and Fermi Energy,” Optics Letters 48, no. 22 (2023): 6012, 10.1364/ol.507991. 37966776

[64] Y. Tang , H. Chen , Y. Li , X.‐Q. Luo , L. Jin , and X.‐L. Wang , “Highly Sensitive and Actively Tunable Chiral Metasurface Enabled by Bound States in the Continuum,” Optics Express 32, no. 22 (2024): 40007, 10.1364/oe.540676. 39573807

