# Supplementary material for: Magnetically Reconfigurable Mode Strong Coupling Toward Tunable Nanophotonic Sensing Devices
Source: Nanophotonics. 2026 Jun 17;15(12):e70174. doi: 10.1002/nap2.70174 (PMC13642554; doi:10.1002/nap2.70174)
Supplement: Supplementary file 1 [file NAP2-15-e70174-s001.pdf]

Supporting Information

Magnetically reconfigurable mode strong coupling towards tunable nanophotonic sensing devices

Runmin Liu, Shengyi Wang, Liangui Deng, Hua Ge, Qiu Wang*, and Bowen Jia*

*Corresponding authors: wangqiu@whut.edu.cn; jiabowen@whut.edu.cn

S1 Numerical simulation details

Full-wave simulations were carried out in COMSOL Multiphysics using the frequency-domain finite-element method. The calculation used a single periodic unit cell of the bismuth iron garnet (BIG) dimer metasurface, with the same geometric parameters as those given in the main text. Floquet periodic boundary conditions were applied along the lateral x and y directions, and perfectly matched layers were placed along $\pm z$ to absorb outgoing waves. The structure was excited from the superstrate side by a normally incident TM-polarized plane wave propagating along the $-z$ direction, with the electric field polarized along x. The transmission was obtained by integrating the output power flux and normalizing it to the incident power.

The SiO_2 substrate was treated as lossless with refractive index $n_{\mathrm{s}} = 1.47$. Under an external static magnetic field applied along the x direction, BIG was described by the transverse magneto-optical relative permittivity tensor

$$\boldsymbol{\varepsilon} = \begin{pmatrix} \varepsilon_r & 0 & 0 \\ 0 & \varepsilon_r & i\varepsilon_i \\ 0 & -i\varepsilon_i & \varepsilon_r \end{pmatrix}. \tag{S1}$$

The diagonal element was set to $\varepsilon_r = 6.25$ in the near-infrared regime [1–3]. Here, ε_i denotes an effective gyrotropic strength parameter associated with the off-diagonal magneto-optical response of BIG, rather than a direct measure of the external magnetic-field intensity itself. This form of the permittivity tensor is commonly used for transversely magnetized magneto-optical garnets and related magnetoplasmonic structures [4–6]. In particular, previous modeling of Bi-substituted YIG/BIG magneto-optical garnets described the transverse gyrotropy by $\varepsilon_{yz} = iq(M_T)$ and adopted $q(M_T^{\mathrm{sat}}) = 0.1$ at saturated transverse magnetization, based on extrapolated Bi:YIG magneto-optical data [3, 7, 8]. The off-diagonal tensor element is odd under magnetization reversal and is proportional to the local magnetization [3]. Therefore, the upper value $\varepsilon_i = 0.1$ used in this work should be interpreted as a representative saturated gyrotropic response of BIG/Bi:YIG-type magneto-optical garnets, instead of a quantity corresponding to a unique external magnetic-field strength.

Experimentally, the magnetic field required to approach magnetic saturation in BIG depends on the film orientation, magnetic anisotropy, strain state, and growth conditions. For epitaxial BIG films, Adachi *et al.* reported saturation magnetizations of $4\pi M_s \approx 1400$ Gs and 1200 Gs for PLD- and RIBS-grown BIG films, respectively, together with saturated Faraday rotations of 27.8 deg/mm and 26.9 deg/mm at 635 nm [9]. Their magnetization measurements further showed that the films saturate below approximately 600 Oe (~ 0.06 T) in the in-plane magnetic-field geometry, whereas perpendicular saturation requires higher fields above approximately 1.6 kOe (~ 0.16 T) [9]. Thus, the practical field scale associated with a saturated BIG gyrotropic response is typically on the order of several tens of mT to about 0.1–0.2 T, depending on the magnetic geometry. Such fields are readily achievable using standard laboratory permanent magnets or electromagnets. For integrated implementations, local magnetic biasing using micromagnets or microcoils is in principle feasible.

The zero-field case corresponds to $\varepsilon_i = 0$, while finite-field calculations used $0 < \varepsilon_i \leq 0.1$ [3, 10]. Unless otherwise stated, the representative strong-coupling and sensing calculations used $\delta = 0.34$, $h = 339$ nm, and $\varepsilon_i = 0.1$. Resonance wavelengths were extracted from spectra sampled around the near-infrared resonance window with a wavelength step of 0.1 nm.

S2 Mechanism of the magneto-structurally enabled MS-BIC

To clarify the physical origin of the MS-BIC, we performed additional band-diagram, field-distribution, control-transmission, and Q-factor analyses by separately examining the roles of magneto-optical coupling and structural asymmetry. The external static magnetic field is applied along the x direction. Under this configuration, the relative permittivity tensor of BIG is written as

$$\boldsymbol{\varepsilon} = \begin{pmatrix} \varepsilon_r & 0 & 0 \\ 0 & \varepsilon_r & i\varepsilon_i \\ 0 & -i\varepsilon_i & \varepsilon_r \end{pmatrix}.$$

Therefore, the electric displacement components are

$$D_x = \varepsilon_0 \varepsilon_r E_x,$$

$$D_y = \varepsilon_0(\varepsilon_r E_y + i\varepsilon_i E_z),$$

$$D_z = \varepsilon_0(-i\varepsilon_i E_y + \varepsilon_r E_z).$$

The magneto-optical contribution to the displacement field can thus be written as

$$\Delta\mathbf{D}_{\mathrm{MO}} = \varepsilon_0 \begin{pmatrix} 0 \\ i\varepsilon_i E_z \\ -i\varepsilon_i E_y \end{pmatrix}.$$

This expression shows that the magneto-optical perturbation is not an ordinary scalar refractive-index perturbation. Instead, it directly couples the E_y and E_z components with a quadrature phase factor. As a result, finite ε_i modifies the electric-field component composition of the mode through magneto-optically induced E_y–E_z mixing.

Figure S1(a,b) shows the eigenmode band diagrams of the symmetric structure ($\delta = 0$) calculated from eigenfrequency simulations for $\varepsilon_i = 0$ and $\varepsilon_i = 0.1$, respectively. The red symbols highlight the eigenmode branch associated with the MS-BIC, whereas the blue symbols denote other eigenmode branches in the same spectral range. At $\varepsilon_i = 0$, the red branch is not yet an independently observable MS-BIC resonance. Instead, it represents a dark parent eigenmode of the symmetric structure. The nearly unchanged band diagrams in Figure S1(a,b) indicate that the external magnetic field does not generate a new eigenmode branch from the continuum. Rather, the MS-BIC-related branch already exists in the symmetric structure, while the magnetic field modifies its electric-field component composition.

The corresponding eigenmode field distributions at the Γ point are shown in Figure S1(c). The dominant $\mathrm{Re}(E_z)$ profiles for $\varepsilon_i = 0$ and $\varepsilon_i = 0.1$ are nearly identical, indicating that the main modal skeleton of the red-marked branch is preserved under the external magnetic field. In contrast, the $|E_y|$ distribution is modified when ε_i is finite. This behavior is consistent with the tensor analysis above: the off-diagonal gyrotropic terms do not primarily reshape the dominant E_z field pattern, but introduce additional E_y–E_z field-component mixing. Therefore, the role of the external magnetic field is to modify the electric-field component composition of a pre-existing eigenmode branch, rather than to create a completely new eigenmode.

However, magneto-optically induced field-component mixing alone is not sufficient to make the mode visible in the far-field transmission spectrum. As shown in Figure S1(d), for the symmetric structure ($\delta = 0$), no transmission resonance appears either at $\varepsilon_i = 0$ or at $\varepsilon_i = 0.1$.

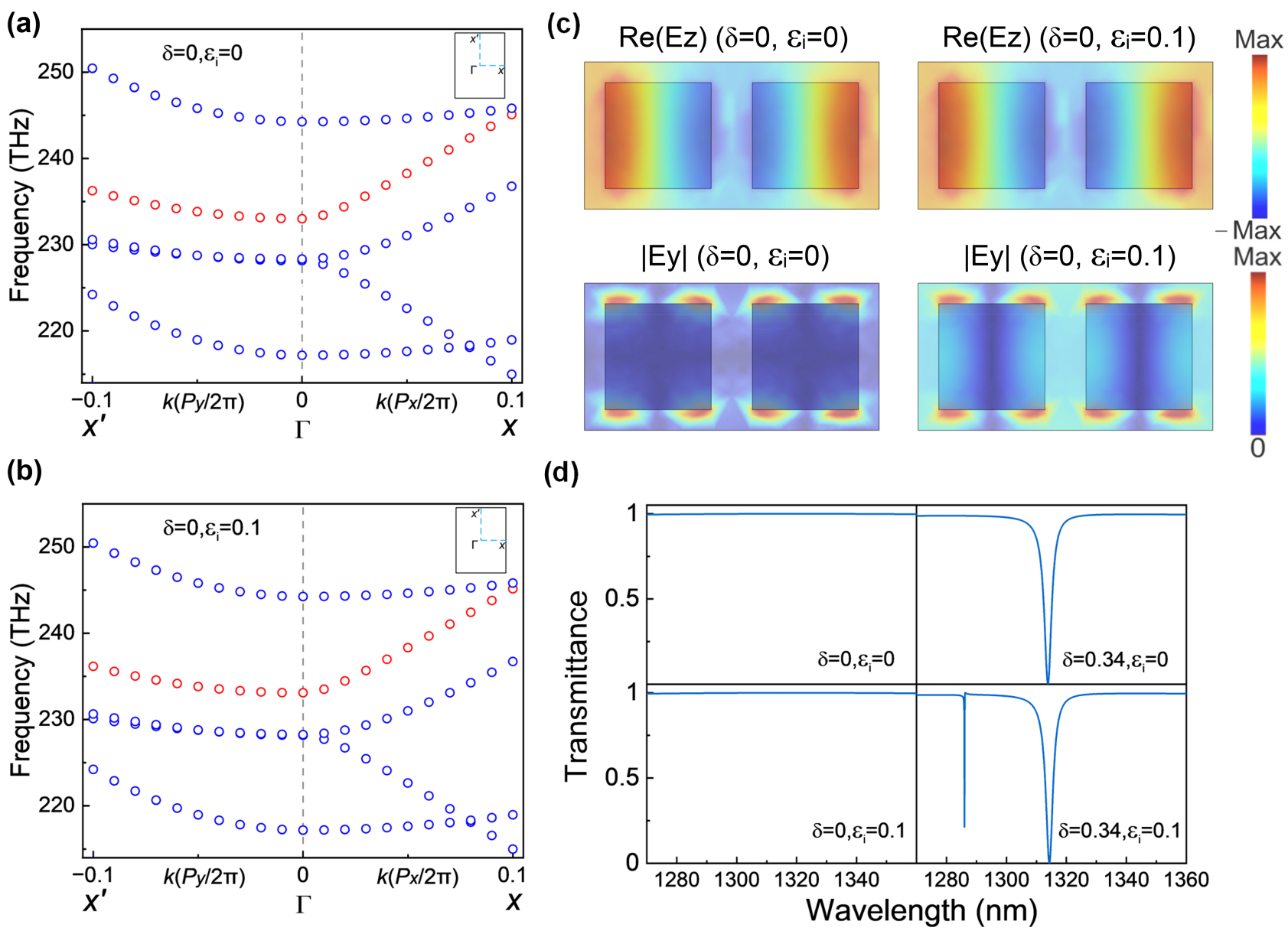

Figure S1. Mechanism analysis of the magneto-structurally enabled MS-BIC. (a,b) Eigenmode band diagrams of the symmetric structure ($\delta = 0$) at (a) $\varepsilon_i = 0$ and (b) $\varepsilon_i = 0.1$. Red and blue symbols denote the MS-BIC-related branch and other eigenmode branches, respectively. (c) Eigenmode field distributions at the Γ point for the red-marked branch, including $\mathrm{Re}(E_z)$ and $|E_y|$ components. (d) Control transmission spectra under different combinations of δ and ε_i, showing that the additional MS-BIC resonance appears only when finite structural asymmetry and finite magneto-optical coupling are simultaneously present.

This means that the spatial symmetry protection of the symmetric dimer remains effective, and the overlap between the red-marked branch and the normally incident radiation channel remains negligible. In other words, although the external magnetic field modifies the internal electric-field component composition of the eigenmode, the mode remains spectrally dark when the structure is spatially symmetric.

When a finite structural asymmetry is introduced ($\delta = 0.34$), the symmetry mismatch between the mode and the radiation channel is relaxed. Consequently, the symmetry-protected structural BIC is converted into a radiative q-BIC, giving rise to a single pronounced resonance at $\varepsilon_i = 0$. Under simultaneous finite structural asymmetry and finite magneto-optical coupling ($\delta = 0.34$, $\varepsilon_i = 0.1$), the magnetically modified dark branch becomes spectrally visible as an additional resonance, resulting in the dual-resonance response. This additional resonance corresponds to the observable form of the MS-BIC.

To further quantify the evolution of the MS-BIC-related branch under the working asymmetric configuration, we calculated the Q factor of the eigenmode near 233 THz as a function of ε_i at fixed $\delta = 0.34$, as shown in Figure S2. This frequency corresponds to the MS-BIC-related branch discussed above. As ε_i increases from 0 to 0.1, the Q factor decreases monotonically by several orders of magnitude. This result indicates that, once structural asymmetry is present, increasing magneto-optical coupling progressively enhances the radiative leakage of the MS-BIC-related branch and drives it away from the high-Q near-BIC limit. Conversely, as ε_i approaches zero at fixed finite δ, the branch tends toward a much higher-Q state and the separate MS-BIC resonance loses its spectral visibility rather than remaining as an independent bright resonance. Over the finite magneto-optical-strength range, the extracted Q factors approximately follow an inverse-quadratic dependence on ε_i, which can be expressed as $Q \propto \varepsilon_i^{-2}$. This behavior is analogous to the characteristic scaling law of quasi-BICs.

Therefore, the Q-factor evolution in Figure S2 provides a quantitative complement to the

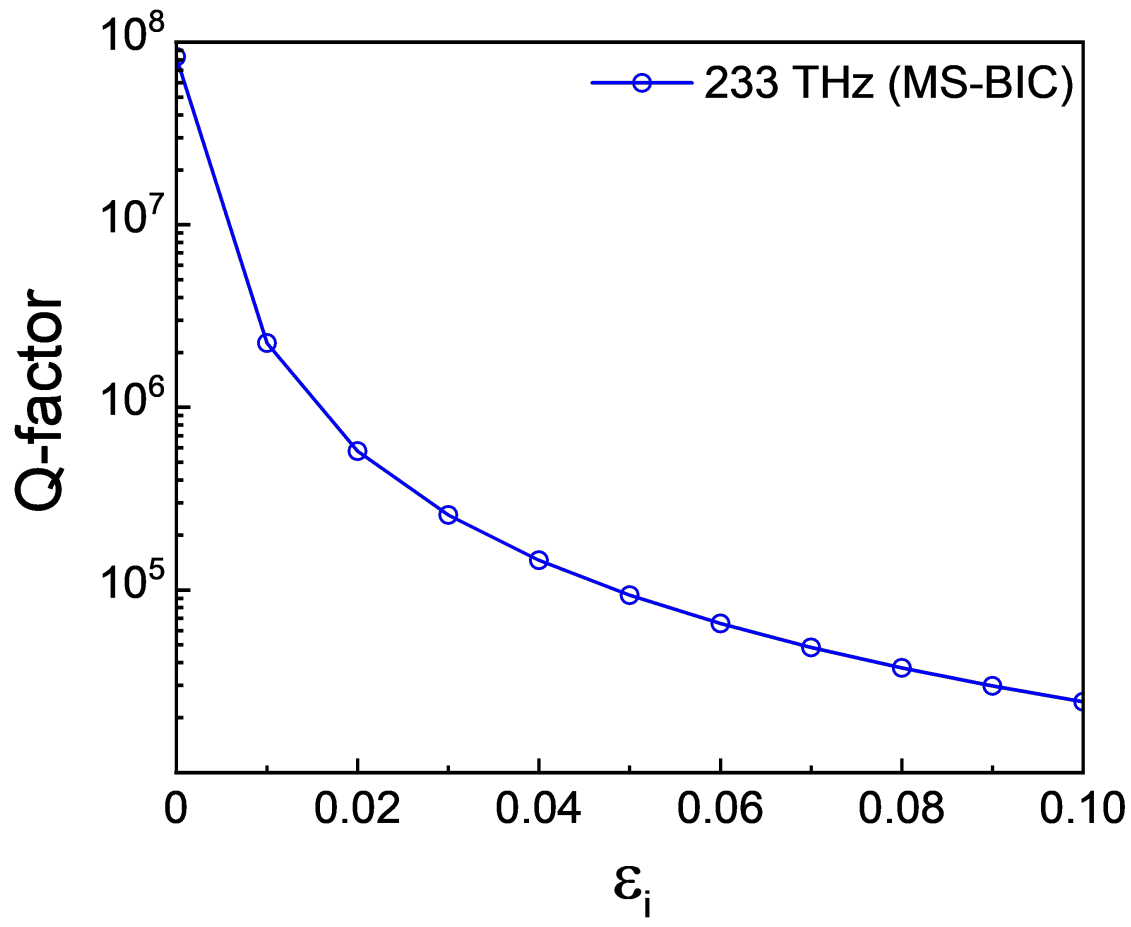

Figure S2. Q-factor evolution of the MS-BIC-related branch at fixed structural asymmetry. The Q factor of the eigenmode branch near 233 THz is plotted as a function of ε_i for $\delta = 0.34$. The monotonic decrease of Q indicates enhanced radiative leakage under increasing magneto-optical coupling.

control spectra in Figure S1(d). The control spectra show whether the resonance is observable in the transmission spectrum, while the Q-factor analysis shows how the radiative leakage of the MS-BIC-related eigenmode branch evolves with the magneto-optical strength under fixed structural asymmetry. Together, these results demonstrate that finite structural asymmetry and finite magneto-optical coupling are both required to produce the observable MS-BIC resonance.

These analyses show that the external magnetic field and structural asymmetry play distinct but complementary roles. The external magnetic field modifies the electric-field component composition of the MS-BIC-related branch through E_y–E_z coupling and, under finite structural asymmetry, controls its radiative leakage and spectral prominence. In contrast, finite structural asymmetry breaks the spatial symmetry protection and provides the necessary radiative leakage channel for spectral observation. Therefore, the observable MS-BIC resonance arises from the combined action of magneto-optical coupling and structural symmetry breaking, rather than from the magnetic field alone.

S3 Multipole decomposition

A Cartesian multipole decomposition was performed to identify the radiation channels associated with the resonant modes. The induced polarization current density inside the BIG resonators was calculated as

$$\mathbf{J}(\mathbf{r},\omega) = -i\omega\varepsilon_0 \left[\boldsymbol{\varepsilon}(\mathbf{r},\omega) - \mathbf{I}\right] \mathbf{E}(\mathbf{r},\omega), \tag{S2}$$

where $\mathbf{I}$ is the identity tensor. All volume integrals were evaluated over the two BIG nanoblocks in one unit cell. Following the current-density formulation for toroidal excitations [11, 12], the electric dipole (ED), magnetic dipole (MD), and toroidal dipole (TD) moments were obtained from

$$\mathbf{p}(\omega) = \frac{1}{-i\omega} \int_V \mathbf{J}(\mathbf{r},\omega)\, \mathrm{d}^3\mathbf{r}, \tag{S3}$$

$$\mathbf{m}(\omega) = \frac{1}{2c} \int_V \mathbf{r} \times \mathbf{J}(\mathbf{r},\omega)\, \mathrm{d}^3\mathbf{r}, \tag{S4}$$

$$\mathbf{T}(\omega) = \frac{1}{10c} \int_V \left[(\mathbf{r}\cdot\mathbf{J}(\mathbf{r},\omega))\,\mathbf{r} - 2r^2\mathbf{J}(\mathbf{r},\omega)\right]\, \mathrm{d}^3\mathbf{r}, \tag{S5}$$

where $\mathbf{r}$ is measured from the geometric center of the unit cell, $r = |\mathbf{r}|$, and c is the speed of light in vacuum.

The electric quadrupole (EQ) and magnetic quadrupole (MQ) tensors were calculated as

$$Q^{(e)}_{\alpha\beta}(\omega) = \frac{1}{-i\,2\omega}\int_V \left[r_\alpha J_\beta(\mathbf{r},\omega) + r_\beta J_\alpha(\mathbf{r},\omega) - \frac{2}{3}\left(\mathbf{r}\cdot\mathbf{J}(\mathbf{r},\omega)\right)\delta_{\alpha\beta}\right] \mathrm{d}^3\mathbf{r}, \tag{S6}$$

$$Q^{(m)}_{\alpha\beta}(\omega) = \frac{1}{3c}\int_V \left[\left(\mathbf{r}\times\mathbf{J}(\mathbf{r},\omega)\right)_\alpha r_\beta + \left(\mathbf{r}\times\mathbf{J}(\mathbf{r},\omega)\right)_\beta r_\alpha\right] \mathrm{d}^3\mathbf{r}, \tag{S7}$$

where $\alpha, \beta \in \{x, y, z\}$ and $\delta_{\alpha\beta}$ is the Kronecker delta. The complex multipole amplitudes were exported as functions of wavelength, and their squared moduli were normalized to compare the relative modal contributions.

S4 Coupled-oscillator model and parameter extraction

The interaction between the structural quasi-bound state in the continuum (q-BIC) and the magneto-structurally induced q-BIC (MS-BIC) was analyzed using a two-mode non-Hermitian coupled-oscillator model. In the energy representation, the Hamiltonian is

$$\hat{H} = \begin{pmatrix} E_\mathrm{q} - i\Gamma_\mathrm{q}/2 & g \\ g & E_\mathrm{MS} - i\Gamma_\mathrm{MS}/2 \end{pmatrix}, \tag{S8}$$

where E_q and E_MS are the bare-mode energies of the structural q-BIC and MS-BIC, respectively, Γ_q and Γ_MS are their full widths at half maximum (FWHM) in energy units, and g is the coherent coupling strength. Resonance wavelengths were converted to photon energies through

$$E = \frac{hc}{\lambda}. \tag{S9}$$

The bare-mode linewidths were extracted from the corresponding parent-mode spectra in the spectrally separated regime used for bare-mode identification and were then converted into the energy domain, so that the coupling strength and dissipation rates were evaluated on the same scale.

In the lossless limit, the detuning between the two parent modes is

$$\Delta = E_\mathrm{q} - E_\mathrm{MS}. \tag{S10}$$

The hybrid eigenenergies are then

$$E_\pm = \frac{E_\mathrm{q} + E_\mathrm{MS}}{2} \pm \frac{1}{2}\sqrt{\Delta^2 + 4g^2}, \tag{S11}$$

where E_+ and E_- denote the higher- and lower-energy eigenvalues, respectively. The upper-branch (UB) and lower-branch (LB) labels are assigned by tracking the two hybrid resonance branches in the wavelength-domain dispersion after applying the conversion $E = hc/\lambda$; thus, the branch labels follow the spectral evolution used in the main text rather than only the $+/-$ sign in Eq. (S11). The Rabi-like splitting is defined as

$$\hbar\Omega = |E_\mathrm{UB} - E_\mathrm{LB}|. \tag{S12}$$

The linewidth-based strong-coupling condition is written as

$$\hbar\Omega > \frac{\Gamma_\mathrm{q} + \Gamma_\mathrm{MS}}{2}. \tag{S13}$$

This criterion was used to assess whether the observed branch separation exceeds the average dissipation scale of the two parent resonances.

The normalized eigenstates are expressed as

$$\begin{aligned}\psi_{\mathrm{UB}} &= \alpha_{\mathrm{UB}}\,|\mathrm{q}\rangle + \beta_{\mathrm{UB}}\,|\mathrm{MS}\rangle,\\ \psi_{\mathrm{LB}} &= \alpha_{\mathrm{LB}}\,|\mathrm{q}\rangle + \beta_{\mathrm{LB}}\,|\mathrm{MS}\rangle,\end{aligned} \tag{S14}$$

with $|\alpha|^2 + |\beta|^2 = 1$. In the lossless approximation, the Hopfield fractions are

$$\begin{aligned}|\alpha_{\mathrm{UB}}|^2 &= \frac{1}{2}\left(1 + \frac{\Delta}{\sqrt{\Delta^2 + 4g^2}}\right), \quad |\beta_{\mathrm{UB}}|^2 = 1 - |\alpha_{\mathrm{UB}}|^2,\\ |\alpha_{\mathrm{LB}}|^2 &= \frac{1}{2}\left(1 - \frac{\Delta}{\sqrt{\Delta^2 + 4g^2}}\right), \quad |\beta_{\mathrm{LB}}|^2 = 1 - |\alpha_{\mathrm{LB}}|^2.\end{aligned} \tag{S15}$$

These fractions were used to quantify the structural q-BIC and MS-BIC contents of the two hybrid branches across the anticrossing region.

Assuming independent decay channels, the hybrid linewidths can be approximated by Hopfield-weighted averages,

$$\begin{aligned}\Gamma_{\mathrm{UB}} &\approx |\alpha_{\mathrm{UB}}|^2\Gamma_{\mathrm{q}} + |\beta_{\mathrm{UB}}|^2\Gamma_{\mathrm{MS}},\\ \Gamma_{\mathrm{LB}} &\approx |\alpha_{\mathrm{LB}}|^2\Gamma_{\mathrm{q}} + |\beta_{\mathrm{LB}}|^2\Gamma_{\mathrm{MS}}.\end{aligned} \tag{S16}$$

S5 Sensing simulation and effective-index model

The refractive-index sensing simulations were performed at the strong-coupling operating point, with $\delta = 0.34$, $h = 339$ nm, and $\varepsilon_i = 0.1$. During the sensing simulation, the metasurface geometry and magnetic-field strength were kept fixed, and only the refractive index of the surrounding medium was varied.

The gas/volatile organic compound (VOC) sensing configuration was modeled using an effective-index approach. In practical vapor sensing, the metasurface can be combined with a low-index porous functional layer, such as an aerogel-like silica coating, which captures analyte molecules through adsorption and capillary condensation. Silica aerogel and aerogel-like mesoporous silica coatings can provide ultralow refractive indices approaching that of air, depending on their density and porosity [13]. Therefore, instead of explicitly resolving the pore geometry and adsorption process, the analyte-loaded region above the metasurface was treated as a homogeneous and lossless superstrate with an effective refractive index n_{eff}.

Here, n_{eff} should not be interpreted as the bulk refractive index of the gas alone. It represents the effective optical environment of the porous overlayer after analyte uptake, as sampled by the near field of the metasurface. The baseline value $n_{\mathrm{eff},0} = 1.01$ was chosen as a representative refractive index for a highly porous, nearly air-like coating, rather than as a fixed material constant of a specific aerogel sample. An increase in analyte loading then corresponds to an increase in n_{eff}, which produces measurable shifts of the UB and LB resonances.

For each value of n_{eff} from 1.01 to 1.15, a transmission spectrum $T(\lambda)$ was calculated using the same electromagnetic model described in Section S1. The two resonance dips were tracked as the UB and LB according to their spectral continuity. Their resonance wavelengths, λ_{UB} and λ_{LB}, were extracted from the local transmission minima. The refractive-index sensitivity was obtained from the slope of the fitted resonance wavelength,

$$S_j = \frac{\mathrm{d}\lambda_j}{\mathrm{d}n_{\mathrm{eff}}} \approx \frac{\Delta\lambda_j}{\Delta n_{\mathrm{eff}}}, \qquad j \in \{\mathrm{UB}, \mathrm{LB}\}. \tag{S17}$$

For the linewidth analysis, the full width at half maximum (FWHM) of each hybrid resonance was extracted from the corresponding simulated transmission dip in the wavelength domain. The

same linewidth-extraction procedure was applied consistently to the UB and LB resonances for all effective refractive indices. The figure of merit was then calculated as

$$\mathrm{FOM} = \frac{S}{\Delta\lambda_{\mathrm{FWHM}}}, \tag{S18}$$

where S is the refractive-index sensitivity and $\Delta\lambda_{\mathrm{FWHM}}$ is the linewidth of the corresponding resonance dip.

The branch separation used for the differential readout was obtained directly from the extracted resonance wavelengths,

$$\Delta\lambda_{\mathrm{split}} = |\lambda_{\mathrm{UB}} - \lambda_{\mathrm{LB}}|\,. \tag{S19}$$

The magnetic reconfiguration of the sensing response was simulated separately under a fixed analyte state. In that calculation, n_{eff} was kept constant at 1.05, corresponding to the representative ethanol-vapor condition used in the main text, while ε_i was swept from 0.02 to 0.10. Any shift of the UB and LB resonances in this calculation is therefore attributed to the change in magneto-optical strength rather than to analyte loading.

A simple estimate of n_{eff} under analyte uptake can be obtained using the Lorentz–Lorenz mixing rule. The refraction function is

$$R(n) = \frac{n^2 - 1}{n^2 + 2}. \tag{S20}$$

For a porous film with porosity ϕ and liquid-filling fraction η inside the voids, the effective refraction function is approximated as [14–16]

$$R_{\mathrm{eff}} \approx (1-\phi)R(n_{\mathrm{skel}}) + \phi\left[(1-\eta)R(n_{\mathrm{air}}) + \eta R(n_{\mathrm{liq}})\right]. \tag{S21}$$

Here, n_{skel} is the refractive index of the solid skeleton, $n_{\mathrm{air}} \approx 1.00$, and n_{liq} is the refractive index of the condensed analyte phase. The corresponding effective refractive index is obtained by inverting Eq. (S20),

$$n_{\mathrm{eff}} = \sqrt{\frac{1 + 2R_{\mathrm{eff}}}{1 - R_{\mathrm{eff}}}}. \tag{S22}$$

This model explains why a highly porous scaffold can have a baseline refractive index close to air and why analyte uptake increases the effective refractive index. For example, an empty silica-based porous coating with a very high air fraction can yield n_{eff} close to 1.01, whereas partial replacement of air in the pores by condensed or adsorbed analyte molecules increases R_{eff} and hence n_{eff}.

In the present work, the filling fraction η is not extracted from a specific adsorption isotherm or vapor-loading experiment. It is used only as a phenomenological parameter to illustrate the physical connection between vapor uptake and the increase of the effective refractive index. Since the exact relation between η and the relative vapor pressure depends on the pore size distribution, surface chemistry, temperature, and capillary condensation behavior, the electromagnetic simulations are parametrized directly by n_{eff}. The vapor-pressure percentages shown in the main text should therefore be understood as representative analyte-loading labels, while the quantitative sensing response is evaluated as a function of n_{eff}.

To further examine whether the proposed metasurface can operate in higher-index liquid environments, we performed additional simulations for water and ethanol. In these simulations, the sensing layer thickness was kept at 2 μm, the same as that used in the other sensing simulations. Water and ethanol were represented by refractive indices of $n = 1.33$ and $n = 1.36$, respectively.

As shown in Figure S3(a,b), two resonance features remain observable in both water and ethanol at $\varepsilon_i = 0.1$. The resonance wavelengths appear in different spectral regions for the two liquid environments, indicating that the metasurface response remains sensitive to the surrounding refractive index even in the higher-index regime.

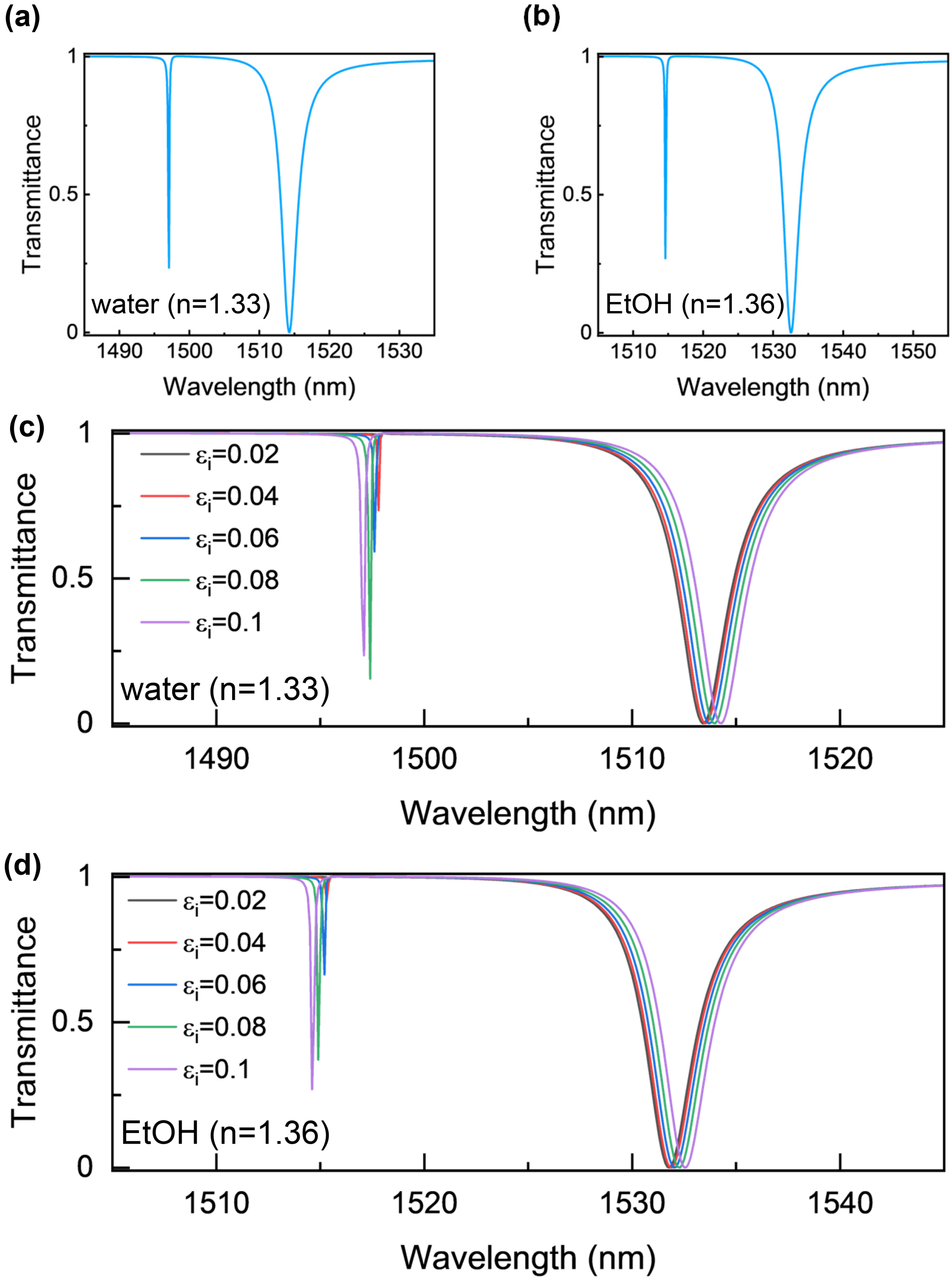

Figure S3. Simulated transmission spectra in higher-index liquid environments. (a,b) Transmission spectra for water ($n = 1.33$) and ethanol ($n = 1.36$) at $\varepsilon_i = 0.1$, respectively. (c,d) Magnetic-field-dependent transmission spectra for water and ethanol as ε_i varies from 0.02 to 0.1. The structural parameters are fixed at $h = 339$ nm and $\delta = 0.34$, and the sensing-layer thickness is 2 μm. The two resonance features remain observable in both liquid environments and can be tuned by the magneto-optical strength.

We further examined the magnetic-field-dependent response in these two liquid environments. As shown in Figure S3(c,d), when the magneto-optical strength ε_i is varied from 0.02 to 0.1, the resonance positions and spectral separation change continuously in both water and ethanol. These results indicate that the resonance features remain detectable and magnetically tunable under higher-index liquid conditions. Therefore, although the sensing performance is more favorable in the low-index porous-coating regime, the proposed structure can still, in principle, provide detectable and magnetically tunable spectral responses in higher-index liquids such as water and ethanol.

References

[1] Enxu Yao, Zhaoxian Su, Yu Bi, Yongtian Wang, and Lingling Huang. Tunable quasi-bound states in the continuum in magneto-optical metasurfaces. *Journal of Physics D: Applied Physics*, 57(37):375104, 2024.

[2] T. Tepper and C. A. Ross. Pulsed laser deposition and refractive index measurement of fully substituted bismuth iron garnet films. *Journal of Crystal Growth*, 255(3–4):324–331, 2003.

[3] Lukáš Halagačka, Mathias Vanwolleghem, Kamil Postava, B. Dagens, and Jaromír Pištora. Coupled mode enhanced giant magnetoplasmonics transverse Kerr effect. *Optics Express*, 21(19):21741–21755, 2013.

[4] V. I. Belotelov, I. A. Akimov, M. Pohl, V. A. Kotov, S. Kasture, A. S. Vengurlekar, A. V. Gopal, D. R. Yakovlev, A. K. Zvezdin, and M. Bayer. Enhanced magneto-optical effects in magnetoplasmonic crystals. *Nature Nanotechnology*, 6(6):370–376, 2011.

[5] Gaspar Armelles, Alfonso Cebollada, Antonio García-Martín, and María Ujué González. Magnetoplasmonics: Combining magnetic and plasmonic functionalities. *Advanced Optical Materials*, 1(1):10–35, 2013.

[6] A. K. Zvezdin and V. A. Kotov. *Modern Magnetooptics and Magnetooptical Materials*. Institute of Physics Publishing, Bristol, UK, 1997.

[7] B. Vertruyen, R. Cloots, J. S. Abell, T. J. Jackson, R. C. da Silva, E. Popova, and N. Keller. Curie temperature, exchange integrals, and magneto-optical properties in off-stoichiometric bismuth iron garnet epitaxial films. *Physical Review B*, 78:094429, 2008.

[8] V. Doormann, J.-P. Krumme, and H. Lenz. Optical and magneto-optical tensor spectra of bismuth-substituted yttrium-iron-garnet films. *Journal of Applied Physics*, 68(7):3544–3553, 1990.

[9] N. Adachi, V. P. Denysenkov, S. I. Khartsev, A. M. Grishin, and T. Okuda. Epitaxial $Bi_3Fe_5O_{12}$(001) films grown by pulsed laser deposition and reactive ion beam sputtering techniques. *Journal of Applied Physics*, 88(5):2734–2739, 2000.

[10] Junqing Li, Zhixu Wu, Dandan Zhang, Yong Sun, Wenxing Liu, and Tianbao Yu. Nonreciprocal toroidal dipole resonance and one-way quasi-bound state in the continuum. *Optics Letters*, 49(5):1313–1316, 2024.

[11] T. Kaelberer, V. A. Fedotov, N. Papasimakis, D. P. Tsai, and N. I. Zheludev. Toroidal dipolar response in a metamaterial. *Science*, 330(6010):1510–1512, 2010.

[12] V. Savinov, V. A. Fedotov, and Nikolay I. Zheludev. Toroidal dipolar excitation and macroscopic electromagnetic properties of metamaterials. *Physical Review B*, 89(20):205112, 2014.

[13] Jieyu Wang, Donald Petit, and Shenqiang Ren. Transparent thermal insulation silica aerogels. *Nanoscale Advances*, 2(12):5504–5515, 2020.

[14] Jifang Tao, Xuerui Wang, Tao Sun, Hong Cai, Yuxiang Wang, Tong Lin, Dongliang Fu, Lennon Lee Yao Ting, Yuandong Gu, and Dan Zhao. Hybrid photonic cavity with metal-organic framework coatings for the ultra-sensitive detection of volatile organic compounds with high immunity to humidity. *Scientific Reports*, 7:41640, 2017.

[15] Nebiyu A. Yebo, Petra Lommens, Zeger Hens, and Roel Baets. An integrated optic ethanol vapor sensor based on a silicon-on-insulator microring resonator coated with a porous ZnO film. *Opt. Express*, 18(11):11859–11866, May 2010.

[16] Neal J Hutchinson, Thomas Coquil, Erik K Richman, Sarah H Tolbert, and Laurent Pilon. Reflectance of surfactant-templated mesoporous silica thin films: Simulations versus experiments. *Thin Solid Films*, 518(8):2134–2140, 2010.
